# Dynamic responses of striatal cholinergic interneurons control behavioral flexibility

**DOI:** 10.1126/sciadv.adn2446

**Published:** 2024-12-18

**Authors:** Zhenbo Huang, Ruifeng Chen, Matthew Ho, Xueyi Xie, Himanshu Gangal, Xuehua Wang, Jun Wang

**Affiliations:** Department of Neuroscience and Experimental Therapeutics, College of Medicine, Texas A&M University Health Science Center, Bryan, TX, USA.

## Abstract

Striatal cholinergic interneurons (CINs) are key to regulating behavioral flexibility, involving both extinguishing learned actions and adopting new ones. However, the mechanisms driving these processes remain elusive. In this study, we initially demonstrate that chronic alcohol consumption disrupts the burst-pause dynamics of CINs and impairs behavioral flexibility. We next aimed to elucidate the mechanisms by which CIN dynamics control behavioral flexibility. We found that extinction learning enhances acetylcholine (ACh) release and that mimicking this enhancement through optogenetic induction of CIN burst firing accelerates the extinction process. In addition, we demonstrate that disrupting CIN pauses via continuous optogenetic stimulation reversibly impairs the updating of goal-directed behaviors. Overall, we demonstrate that CIN burst firing, which increases ACh release, promotes extinction learning, aiding the extinguishment of learned behaviors. Conversely, CIN firing pauses, which lead to ACh dips, are crucial for reversal learning, facilitating the adaptation of new actions. These findings shed light on how CIN dynamics regulate behavioral flexibility.

## Teaser

Striatal cholinergic interneurons dynamically control the extinction and updating of goal-directed learning.

## INTRODUCTION

Behavioral flexibility, the ability of an organism to adapt its behavior to changing circumstances or environmental cues, is critical for species survival (*1*). Central to this capability is the basal ganglia, which orchestrates the selection and implementation of contextually appropriate responses based on expected outcomes in conjunction with the prefrontal cortex (*2*–*4*). Within the basal ganglia, the striatum, which predominantly receives cognitive inputs from the cerebral cortex and sensory information from the thalamus, stands out as an integrator for voluntary action initiation, execution, and modulation (*5*–*7*). Acetylcholine (ACh) in the dorsomedial striatum (DMS) has been implicated in behavioral flexibility (*8*, *9*). In addition, further studies have identified the cholinergic interneurons (CINs) in the posterior DMS as crucial for behavioral flexibility (*10*–*12*). Our previous study demonstrated that chronic alcohol consumption impairs behavioral flexibility by suppressing thalamostriatal excitation of CINs, yet the precise mechanisms through which CINs regulate behavioral flexibility remain elusive (*13*).

Although CINs constitute a mere 1 to 2% of the striatal cell population, their extensive terminal fields pervade the entire striatum (*14*). CINs provide a major source of ACh to the striatum and modulate striatal output by regulating other types of striatal neurons (*15*–*17*). CINs were shown to correspond to putative “tonically active neurons” (TANs) in early in vivo studies of primates (*18*). They were initially thought to have minimal behavioral state modulation due to their consistent firing during movement (*19*). Subsequent research, however, revealed their responsiveness to reward cues, characterized by a “pause response” after brief burst firing or before “rebound” firing (*16*, *20*–*23*). These CIN dynamic responses to motivationally salient stimuli coincide with phasic changes in dopamine neuron activity (*23*), and increasing literature suggests that ACh and dopamine systems interact during reward processing within the striatum (*18*, *23*–*25*). However, the details of CIN dynamics and their significance across behavioral contexts remain largely elusive.

The mechanisms of CIN dynamic responses are not entirely clear, but multiple hypotheses have been proposed (*26*, *27*). Evidence suggests that the activation of thalamic inputs can induce a burst-pause firing pattern in CINs (*21*, *28*). The intralaminar thalamic nuclei, especially the parafascicular nucleus (PfN), provide major excitatory inputs to CINs (*29*, *30*). An in vivo study with anesthetized rats showed that electrical stimulation of the thalamus could induce pauses in CIN firing (*21*). A primate study also indicated that thalamic inputs were necessary for the pause response of TANs, as local inhibition of thalamic activity by a γ-aminobutyric acid type A receptor agonist, muscimol, attenuated these pauses (*31*). It has been proposed that these pauses provide a time window during which corticostriatal synaptic plasticity can occur (*32*). A recent study demonstrated that the pauses in CIN firing were required to induce corticostriatal plasticity (*33*). However, the behavioral implications of CIN dynamic responses are still unclear, and thus, our study aims to bridge this knowledge gap. We used extinction and reversal learning tasks, which are conducted after initial operant conditioning and both require the suppression of previously learned contingencies, to examine the role of CINs in behavioral flexibility. In addition, reversal learning also involves the acquisition of new contingencies. Using these paradigms, we found that while CIN burst firing accelerates the extinction of acquired behaviors, the pauses in CIN firing prove essential for updating goal-directed learning.

## RESULTS

### Chronic alcohol consumption impairs CIN burst-pause dynamics and behavioral flexibility

**Fig. 1.**
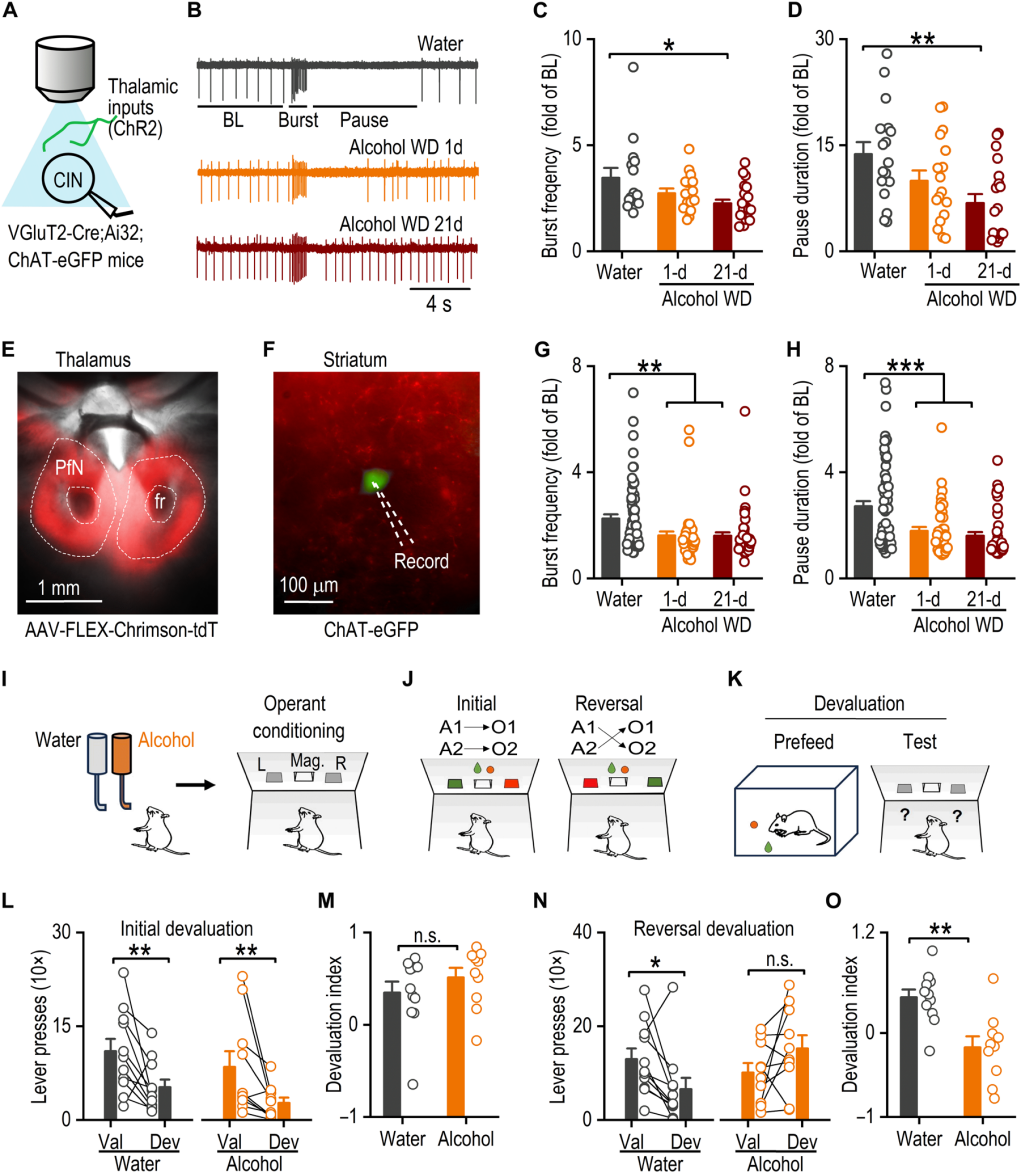
Excessive alcohol intake reduces thalamically evoked burst-pause response in DMS CINs and impairs reversal of operant conditioning. (**A**) Schematic of light stimulation of channelrhodopin-2 (ChR2)–expressing thalamic inputs and recording of CINs. (**B**) Sample traces from cell-attached recording of CINs in response to optical stimulation of thalamic inputs (470 nm, 10 Hz, 10 pulses). (**C**) Bar graph of burst frequency expressed as fold change of baseline firing frequency. *P < 0.05. (**D**) Bar graph of pause duration expressed as fold change of baseline interspike intervals. **P < 0.01. (**E**) Sample image showing tdTomato fluorescence in the PfN. (**F**) Sample image of tdTomato fluorescence and ChAT-eGFP neuron in the striatum. (**G**) Same as (C) with optical stimulation of 590 nm, 20 Hz, 20 pulses. **P < 0.01. (**H**) Same as (D). ***P < 0.001. (**I**) Schematic of alcohol treatment and behavioral training. (**J**) Reversal learning behavioral paradigm. (**K**) Schematic of the devaluation test. (**L**) Both water and alcohol groups pressed the devalued (DeV) lever significantly less than the valued (Val) lever after initial learning; **P < 0.01. (**M**) Devaluation index revealed no significant difference between groups; *P* = 0.31. (**N**) Postreversal learning devaluation. *P < 0.05 (water); *P* = 0.09 (alcohol). (**O**) The alcohol group had a significantly lower devaluation index than the water group; **P < 0.01. Mixed-model analysis of variance (ANOVA) was followed by a simple effects test for (L) and (N); unpaired *t* test for (M) and (O); *n* = 11 rats (water) and 10 rats (alcohol). One-way ANOVA followed by Tukey post hoc test for (C) and (D): *n* = 15 neurons from five mice (water 15/5), (alcohol WD 1-d 16/5), and (alcohol WD 21-d 22/5); for (G) and (H): (water 65/10), (alcohol WD 1-d 41/7), and (alcohol WD 21-d 44/7). Mag., magazine; L, left lever; R, right lever; BL, baseline; WD, withdrawal; n.s., not significant; fr, fasciculus retroflexus; d, day.

Our previous study revealed that chronic alcohol intake and withdrawal led to a long-lasting reduction of thalamostriatal inputs to DMS CINs and the decrease of CIN pause response induced by optogenetic stimulation (*13*). However, it remains unclear whether chronic alcohol consumption directly alters thalamically induced CIN burst-pause dynamics. Given the crucial role of thalamic inputs in triggering these dynamics in CINs (*28*), we hypothesized that chronic alcohol exposure may disrupt CIN dynamics by altering thalamostriatal transmission. To test this hypothesis, we examined the effect of long-term alcohol consumption on CIN burst-pause dynamics using VGluT2-Cre;Ai32;ChAT-eGFP mice (Fig. 1A). Previous studies in VGluT2-Cre mice have shown that VGluT2-expressing inputs to the striatum primarily originate from the thalamus (*34*, *35*). Mice were trained to consume 20% alcohol for 8 weeks using an intermittent-access two-bottle choice drinking procedure (*13*, *36*, *37*). Striatal slices were prepared 1 day or 21 days after the last alcohol exposure, and cell-attached recordings of CINs were conducted in response to optical stimulation of thalamic inputs (Fig. 1B). Notably, burst responses to thalamic stimulation gradually decreased as alcohol withdrawal progressed [Fig. 1C; *F*_(2,50)_ = 4.65, *P* < 0.05]. Similarly, the pause responses of CINs showed a significant reduction, with shortened pause duration over time [Fig. 1D; *F*_(2,50)_ = 5.67, *P* < 0.01]. These findings were further confirmed using whole-cell current-clamp recordings (fig. S1). To validate the alcohol-associated suppression of thalamically induced burst-pause responses, we infused AAV-FLEX-Chrimson-tdT into the PfN of VgluT2-Cre;ChAT-eGFP mice (Fig. 1E). CINs were recorded in cell-attached mode (Fig. 1F), and the results were consistent with those observed in the transgenic mice [burst: Fig. 1G; *F*_(2,147)_ = 6.59, *P* < 0.01; pause: Fig. 1H; *F*_(2,147)_ = 12.45, *P* < 0.001]. These findings suggest that chronic alcohol intake impairs thalamically induced CIN burst-pause dynamics.

Next, we investigated the mechanisms underlying these reduced thalamically induced CIN burst-pause responses following chronic alcohol consumption. Our previous work revealed that chronic alcohol intake leads to a persistent reduction in glutamatergic transmission from the PfN to CINs (*13*). In this study, we found that after alcohol consumption, CINs exhibited decreased AMPA-induced currents (fig. S2, A and B), while *N*-methyl-d-aspartate–induced currents remained unchanged (fig. S2, C and D), suggesting altered postsynaptic AMPA receptor function. In addition, we observed an increase in the spontaneous firing of CINs following alcohol consumption and withdrawal (fig. S2E). These changes—reduced AMPA receptor function combined with heightened baseline activity—likely render CINs less responsive to thalamic inputs, leading to the observed reduction in thalamically induced burst-pause dynamics.

Given the critical role of CINs in behavioral flexibility, the alcohol-induced impairment in CIN dynamics could lead to reduced behavioral flexibility. To test this, we measured behavioral flexibility by examining whether chronic alcohol consumption affected reversal learning in operant tasks. Long-Evans rats were exposed to water or 20% alcohol for 8 weeks and then trained for operant conditioning (Fig. 1I). During the initial training, animals learned to press the left lever [action 1 (A1)] for sucrose [outcome 1 (O1); A1➔O1] and press the right lever for food pellets (A2➔O2). In the subsequent reversal learning, pressing the left lever resulted in food pellets (A1➔O2), and pressing the right lever led to sucrose (A2➔O1) (Fig. 1J and fig. S3, A and B). To assess the learning of action-outcome associations, a devaluation test was conducted after each stage of the initial and reversal learning (Fig. 1K). The initial devaluation test showed that both the water and alcohol groups successfully acquired the initial action-outcome associations (Fig. 1L). Statistical analysis indicated a main effect of devaluation [*F*_(1,19)_ = 14.27, *P* < 0.01] with no significant group x devaluation interaction [*F*_(1,19)_ < 0.001, *P* = 0.99]. In addition, there was no significant difference in the devaluation index between groups [Fig. 1M; *t*_(19)_ = −1.04, *P* = 0.31]. However, after reversal learning, a significant group x devaluation interaction was observed [*F*_(1,19)_ = 8.57, *P* < 0.01]. Further simple effect tests revealed that while the water group significantly preferred the valued over devalued lever [Fig. 1N, left; *F*_(1,19)_ = 5.53, *P* < 0.05], the alcohol group did not [Fig. 1N, right; *F*_(1,19)_ = 3.26, *P* = 0.09]. Furthermore, the devaluation index was significantly lower in the alcohol group than in the water control [Fig. 1O; *t*_(19)_ = 3.79, *P* < 0.01]. These results indicate that while water-exposed rats successfully acquired new action-outcome associations after reversal learning, alcohol-exposed rats failed to do so, demonstrating impaired behavioral flexibility. Given that optogenetic enhancement of PfN-to-CIN transmission can rescue alcohol-induced reversal learning deficits (*13*), our results suggest that the reduced thalamically evoked burst-pause dynamics due to chronic alcohol consumption likely contributes to the observed impairment in reversal learning.

### Reversal and extinction learning enhance ACh release in the DMS

**Fig. 2.**
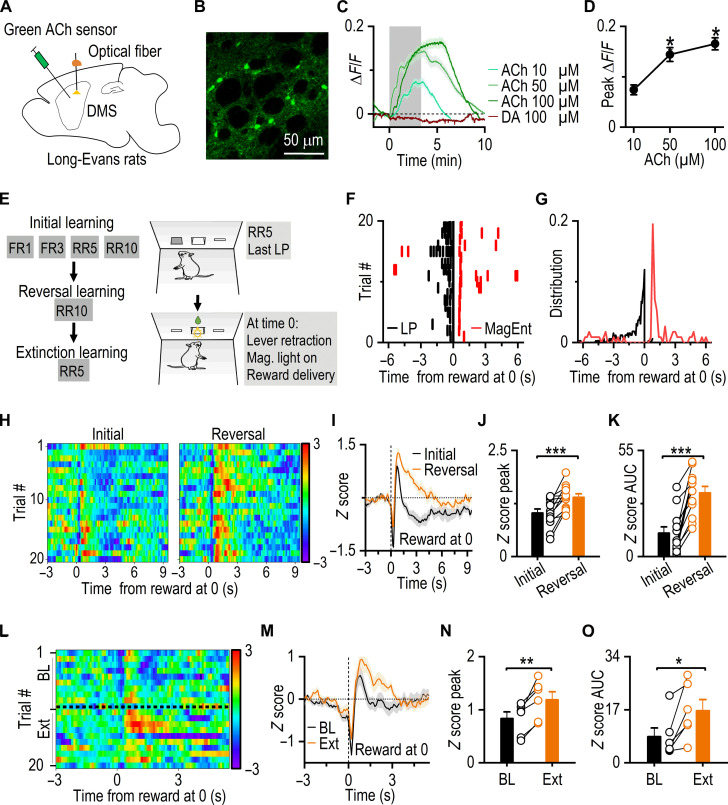
Reversal and extinction learning enhance ACh release. (**A**) Schematic illustrating virus infusion and fiber implantation. (**B**) Sample image of AAV-iAChSnFR expression in the DMS. (**C**) Validation of iAChSnFR sensor using ex vivo confocal imaging. (**D**) Dose-response of ACh-induced changes in iAChSnFR signals. *P < 0.05 by paired *t* test compared to 10 μM, *n* = 6 ROIs (regions of interest) from two rats. (**E**) Behavioral training paradigm and schematic of events at time 0 (the start of reward delivery). (**F**) A representative behavioral plot for lever press (LP) and magazine entry (MagEnt) in a RR5 session. (**G**) Behavioral distribution plot for (F). (**H**) In vivo measurements of ACh signals in the DMS. The heatmap showed ACh signals during the first reversal session, which consisted of initial learning in the first half session and reversal learning in the second half. (**I**) Representative traces of ACh signals for the first reversal session. (**J** and **K**) Summary data quantifying the peak and the AUC of ACh sensor fluorescence signals; ***P < 0.001 by paired *t* test, *n* = 6 sucrose reversal sessions + 6 food reversal sessions from 6 rats. (**L**) In vivo measurements of ACh release during baseline (BL) and extinction (Ext) training within the same session. (**M**) Representative ACh signal traces for BL and Ext. (**N** and **O**) Greater ACh signal peaks and AUC during Ext than BL; *P < 0.05 and **P < 0.01 by paired *t* test, *n* = 6 rats. FR, fixed ratio; RR, random ratio.

Following our findings that chronic alcohol consumption impairs CIN burst-pause dynamics and behavioral flexibility, we next investigated how ACh release, governed by CIN firing dynamics, is modulated during key stages of behavioral flexibility. Since both reversal and extinction learning involve the suppression of previously learned behaviors—with reversal learning requiring the additional acquisition of learning new contingencies—we sought to determine whether both of these forms of behavioral flexibility modulate ACh release in the DMS. To directly measure the ACh signal in the DMS during these tasks, we first infused a genetically encoded green ACh sensor, AAV-iAChSnFR (*38*–*40*), into the DMS of Long-Evans rats and verified its expression (Fig. 2, A and B). The sensor responded in a dose-dependent manner to varying ACh concentrations while remaining unresponsive to dopamine, ensuring specificity [Fig. 2, C and D; *F*_(2, 15)_ = 49.64, *P* < 0.001]. The rats were then subjected to a series of operant conditioning tasks, progressing through initial learning, reversal learning, and lastly extinction learning (Fig. 2E). During each trial, the last lever press triggered the delivery of a reward (either a food pellet or sucrose solution), promoting the animals to quickly move to the center magazine to collect their rewards (Fig. 2, F and G). Throughout these tasks, we continuously monitored ACh signals. Aligning the ACh signals with the onset of reward delivery revealed a distinct dip-rebound-dip pattern in ACh levels (Fig. 2, H and I), with the rebound peak coinciding with the magazine-entry peak (fig. S4A). Notably, during the first reversal session, we discovered a pronounced elevation in the ACh rebound signal following reward delivery. Quantitative analysis of peak signals and the area under the curve (AUC) confirmed that reversal learning significantly increased both the magnitude [peak: Fig. 2J; *t*_(11)_ = −4.71, *P* < 0.001] and duration [AUC: Fig. 2K; *t*_(11)_ = −6.61, *P* < 0.001] of ACh rebound in the DMS. These findings suggest that reversal learning elevates ACh levels in the DMS, consistent with recent research linking reversal learning to enhanced burst firing of DMS CINs (*41*).

Given that behavioral flexibility involves both adapting to new contingencies (as in reversal learning) and extinguishing previously learned behaviors, we next examined whether extinction learning, like reversal learning, also increases ACh levels in the DMS. To investigate this, we conducted a modified session that included an extinction task. During the initial 10 trials, the rats pressed levers to receive rewards, establishing a baseline for ACh signals. The subsequent 10 trials involved lever pressing without any reward, representing the extinction phase. Assessment of the signal heatmap revealed that the ACh rebound fluorescence during extinction trials was more intense and prolonged compared to baseline trials (Fig. 2, L and M). Quantitative analysis revealed that extinction led to a significant increase in both the magnitude [peak: Fig. 2N, *t*_(5)_ = −5.13, *P* < 0.01] and duration [AUC: Fig. 2O, *t*_(5)_ = −3.19, *P* < 0.05] of ACh release in the DMS. These findings suggest that extinction learning enhances ACh rebound in the DMS.

### Thalamic input facilitates CIN burst firing and ACh release during extinction learning

**Fig. 3.**
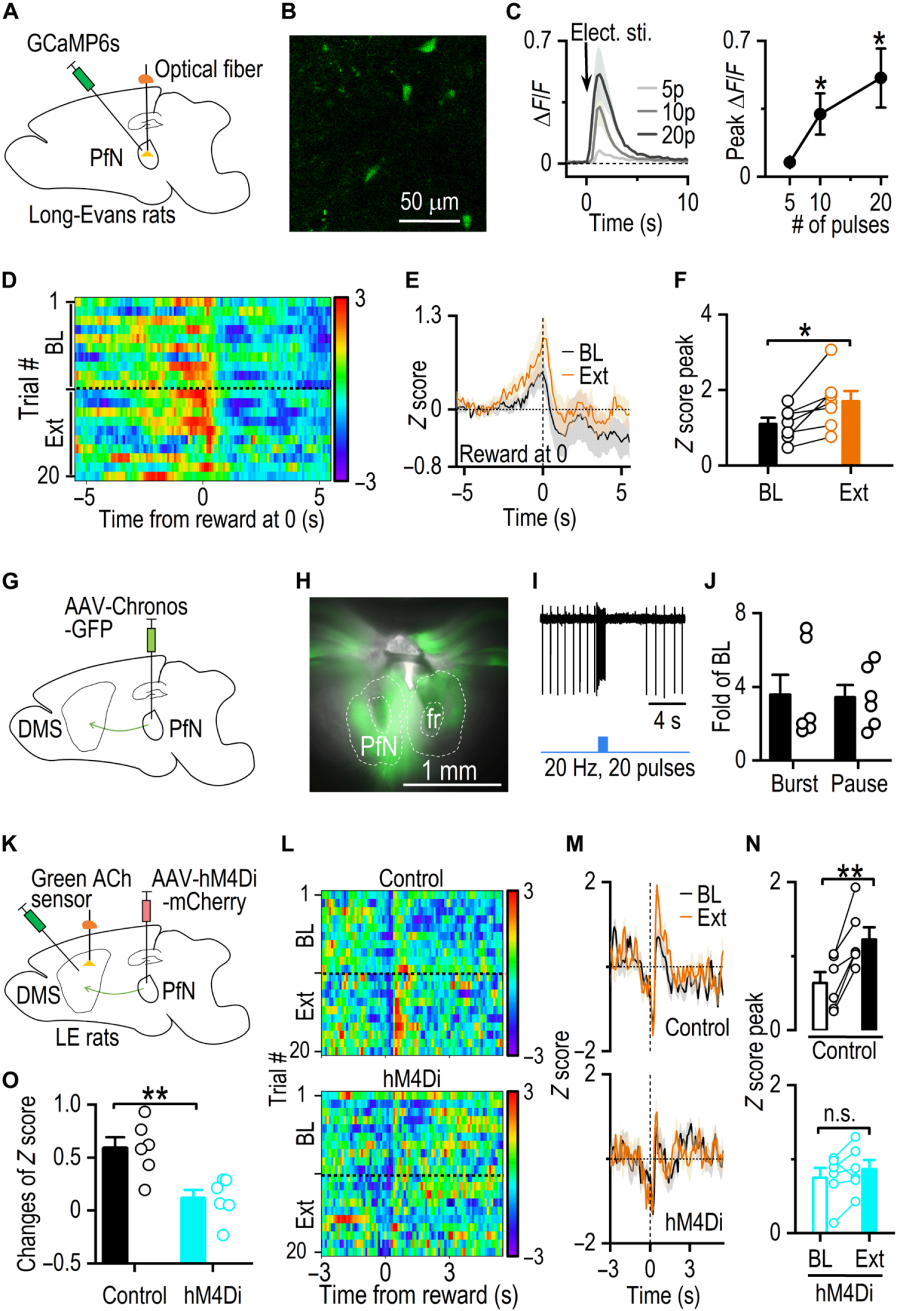
Thalamic input contributes to increased ACh release during extinction. (**A**) Schematic illustrating virus infusion and fiber implantation. (**B**) AAV-GCaMP6s expression in the PfN. (**C**) Validation of AAV-GCaMP6s using ex vivo confocal imaging. Electric stimulation (elect. sti.) caused an increase in GCaMP signals in PfN slices. *P < 0.05 by paired *t* test compared to five pulses, *n* = 6 ROIs from two rats. (**D**) In vivo measurements of GCaMP signal during baseline (BL) and extinction (Ext) training within the same session. (**E**) Representative GCaMP signal traces for BL and Ext. (**F**) Summary data quantifying the peak GCaMP signals; *P < 0.05 by paired *t* test, *n* = 7 rats. (**G**) Schematic illustrating virus infusion. (**H**) Sample image of GFP fluorescence in the PfN. (**I**) Optical stimulation of axon terminals from the PfN induced a burst-pause firing of CINs in the DMS. (**J**) Quantification of burst frequency and pause duration relative to BL, *n* = 6 neurons. (**K**) Schematic illustrating virus infusions and fiber implantation. (**L**) In vivo measurements of ACh release during BL and Ext training within the same session. (**M**) Representative ACh signal traces for BL and Ext. (**N**) Summary data quantifying the peak ACh sensor fluorescence signals; **P < 0.01 by paired *t* test. (**O**) Summary data comparing the changes of peak *Z* score from BL to Ext between two groups; **P < 0.01 by unpaired *t* test. *n* = 6 rats (control) and 6 rats (hM4Di).

Having established that both reversal and extinction learning enhance ACh release in the DMS, we next investigated the mechanisms underlying this modulation. Our rabies virus tracing study identified multiple inputs to CINs, with a particularly high density of input neurons originating from the thalamus (fig. S4, B and C) (*13*). Given the substantial thalamic projections to CINs from the PfN (*13*, *30*, *42*), we hypothesized that the thalamus could be a key excitatory input, facilitating CIN burst firing and subsequent ACh release during extinction learning. To test this hypothesis, we infused AAV-GCaMP6s into the PfN of Long-Evans rats and implanted optical fibers to monitor thalamic activity (Fig. 3A). We confirmed robust expression of AAV-GCaMP6s in the PfN (Fig. 3B) and observed strong responses to electrical stimulations ex vivo (Fig. 3C). Rats were then trained using the same protocol as in Fig. 2E, and fiber photometry was used to record thalamic GCaMP6s signals. We discovered that thalamic activity began to increase ~2 s before reward delivery, peaking at the time of reward delivery (Fig. 3, D and E). Extinction learning significantly elevated the peak amplitude of the GCaMP6s signals compared to the baseline [Fig. 3F; *t*_(6)_ = −3.31, *P* < 0.05]. Similarly, reversal learning also triggered an increase in thalamic activity at the time of reward delivery (fig. S5). These findings suggest that both extinction and reversal learning enhance thalamic activity.

To further establish the connection between the PfN and CINs, we infused AAV-Chronos-GFP into the PfN (Fig. 3, G and H). Patch-clamp recordings confirmed that activating thalamic inputs induced burst-pause firing in DMS CINs (Fig. 3, I and J). To determine whether these thalamic inputs were necessary for the ACh increase during extinction, we infused AAV-hM4Di-mCherry (*36*, *43*) into the PfN and the green ACh sensor, iAChSnFR, into the DMS of rats (Fig. 3K). Optical fibers were implanted in the DMS, and animals were trained to press a lever for sucrose rewards. As expected, extinction training significantly increased ACh release compared to baseline trials [Fig. 3, L to N (top); *t*_(5)_ = −5.60, *P* < 0.01]. However, this increase in ACh release was greatly reduced in the hM4Di group, in which thalamic activity was inhibited by an intraperitoneal injection of the selective hM4Di ligand, deschloroclozapine (DCZ) (*44*, *45*), administered 30 min before the extinction session [Fig. 3, L to N (bottom); *t*_(5)_ = −1.39, *P* = 0.22]. Moreover, the hM4Di group exhibited a significantly lower ACh rise than the control group [Fig. 3O; *t*_(11)_ = 3.58, *P* < 0.01]. We further analyzed ACh signals in the same animals before and after DCZ application during reward sessions (fig. S6). DCZ administration led to a reduction in ACh release compared to a saline injection administrated the previous day, although this decrease did not reach statistical significance [*t*_(5)_ = 2.15, *P* = 0.08]. In contrast, the control group, which received two consecutive saline injections, showed no significant change in ACh release [*t*_(5)_ = 0.35, *P* = 0.74]. These results suggest that thalamic inputs likely play a role in contributing to the ACh rebound observed during reward sessions. Together, these findings demonstrate that thalamic inputs provide a major excitatory drive to CINs during the extinction process, facilitating CIN burst firing and ACh release.

### CIN burst firing accelerates extinction learning

**Fig. 4.**
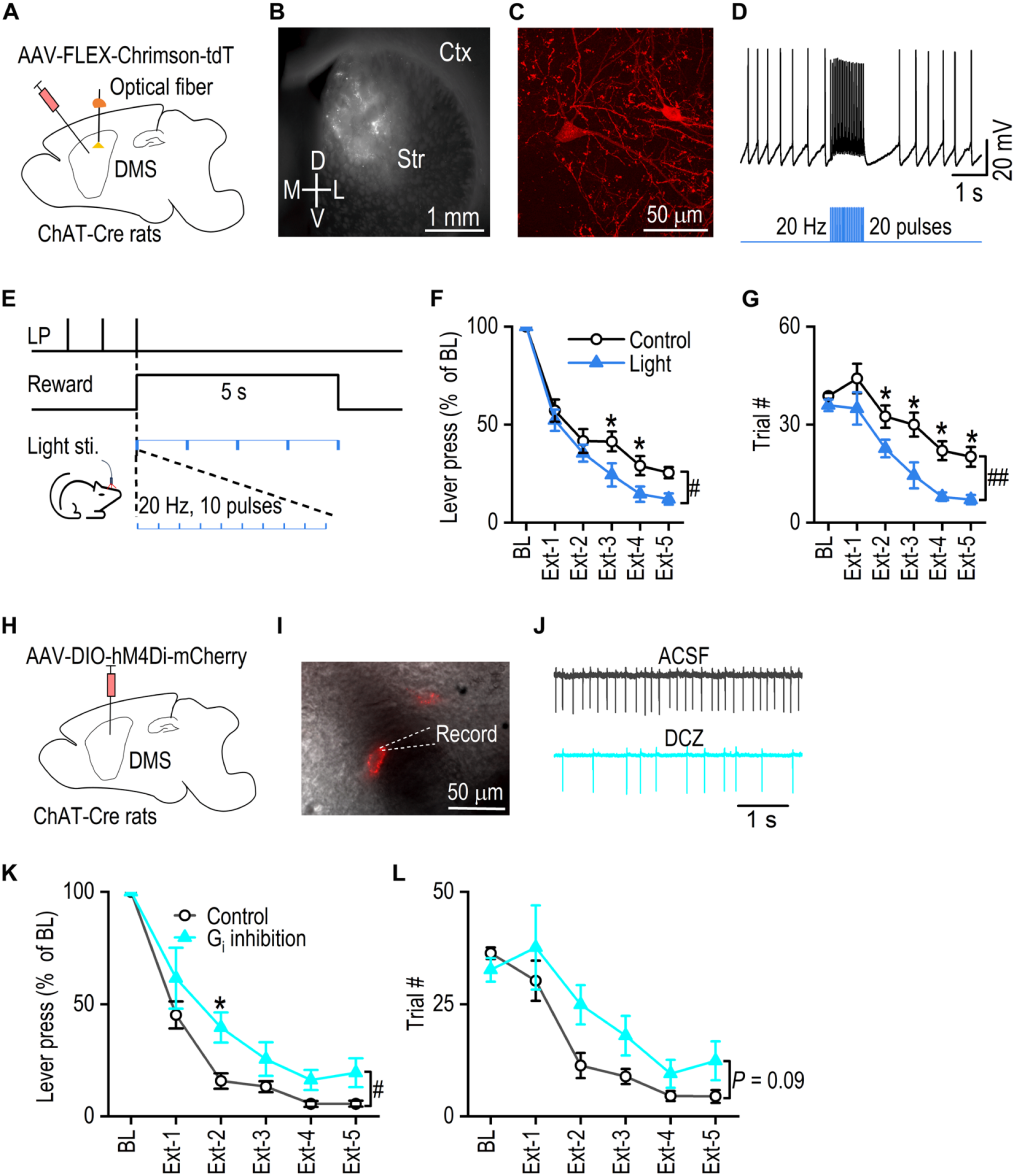
Augmented CIN burst firing accelerates extinction learning. (**A**) ChAT-Cre rats received a bilateral infusion of AAV-FLEX-Chrismon-tdT and optical fiber implantation in the DMS. (**B**) The expression of AAV-FLEX-Chrimson-tdT. Ctx, cortex; Str, striatum; D, dorsal; L, lateral; V, ventral; M, medial. (**C**) Sample confocal image of Chrimson-tdTomato expressed CINs. (**D**) Sample current-clamp recording trace of CIN firing in response to light stimulation. (**E**) The optical stimulation protocol used during the extinction training. (**F**) Lever presses of the light stimulation group were significantly lower than those of the control group during extinction training. The data were normalized to their baseline lever presses. (**G**) The extinction trials were significantly lower in the light group than in the control group. Two-way RM ANOVA followed by Tukey post hoc test, #*P* < 0.05, ##*P* < 0.01, and *P < 0.05, *n* = 13 rats (control) and 13 rats (light). (**H**) Schematic of viral injection: ChAT-Cre rats underwent bilateral infusion of AAV-DIO-hM4Di-mCherry into the DMS. Following this, rats were trained instrumentally to press a lever for sucrose rewards. (**I**) Cell-attached patch clamp recordings were conducted in CINs expressing mCherry to monitor their activity. (**J**) Bath application of DCZ (1 μM) for 10 min significantly inhibited the spontaneous firing of CINs. ACSF, artificial cerebrospinal fluid. (**K**) During extinction training, lever presses in the hM4Di (G_i_) inhibition group (DCZ, 0.2 mg/kg) were significantly higher than those in the control group. Data were normalized to baseline. (**L**) The number of extinction trials was greater in the G_i_ inhibition group compared to the control group. Two-way RM ANOVA followed by Tukey post hoc test, #*P* < 0.05 and *P < 0.05, *n* = 9 rats (control) and 8 rats (G_i_ inhibition).

With thalamic input confirmed as a key driver of CIN burst firing and ACh release during extinction learning, we next investigated whether CIN burst firing is sufficient to accelerate extinction learning. Specifically, we explored whether directly inducing CIN burst firing could enhance the extinction process to further elucidate the role of CIN dynamics in behavioral flexibility. To this end, we bilaterally infused AAV-FLEX-Chrimson-tdT into the DMS of ChAT-Cre rats (Fig. 4A), resulting in robust expression of Chrimson-tdT within the DMS (Fig. 4, B and C). Ex vivo current-clamp recordings confirmed that light stimulation induced a burst-pause response in tdTomato-positive CINs (Fig. 4D). After recovery from surgery, the rats were trained to press levers for rewards using the initial learning procedure described in Fig. 2E (fig. S3C). They then underwent five consecutive daily extinction sessions, each lasting 10 min. During these sessions, the “light” group received five bursts of light stimulation (Fig. 4E), time-locked to the start of the reward delivery period (actual rewards were omitted), whereas the control group received no light stimulation. We chose these stimulation parameters to mimic CIN burst firing in vivo (over 15 Hz for less than 1 s) (*16*, *46*) and induce multiple ACh rises during the reward delivery period, which is expected to greatly affect behavioral extinction. Notably, the light-stimulated group extinguished lever pressing more rapidly, with significantly fewer lever presses than the control group [normalized data: Fig. 4F; group effect: *F*_(1,24)_ = 5.19, *P* < 0.05; session effect: *F*_(1,4)_ = 29.55, *P* < 0.001; group x session effect: *F*_(1,96)_ = 1.02, *P* = 0.40; raw data: fig. S7A]. The light-stimulated group also has a significant decrease in extinction trials [Fig. 4G; group effect: *F*_(1,24)_ = 15.18, *P* < 0.01; session effect: *F*_(1,4)_ = 27.85, *P* < 0.001; group x session effect: *F*_(1,96)_ = 0.47, *P* = 0.76]. These effects were not due to general impacts on motor function or motivation, as CIN burst stimulation did not reduce lever presses or earned rewards when rewards were available, when compared to both the control group (fig. S8, A and B) and their prestimulation behavior (fig. S8C). These findings suggest that CIN burst firing is sufficient to accelerate extinction learning.

To further investigate the role of CINs in extinction learning, we next examined whether inhibiting CIN activity would conversely slow down the extinction process, thereby assessing the necessity of CIN firing in facilitating behavioral flexibility. We used both optogenetic and chemogenetic approaches to inhibit CIN function. Despite successful inhibition of CIN firing ex vivo, our optogenetic approach did not produce a consistent effect on extinction behavior, likely due to a rebound burst firing following light-induced inhibition (fig. S9) (*47*). In our chemogenetic experiment, AAV-DIO-hM4Di-mCherry was bilaterally infused into the DMS of ChAT-Cre rats (Fig. 4H). The hM4Di-mediated inhibition of CINs was confirmed using cell-attached recordings in brain slices (Fig. 4, I and J). The animals were then trained to press a lever for sucrose rewards (fig. S3D), followed by five 30-min extinction sessions. We extended the extinction period in comparison to Fig. 4 (F and G) to accelerate the reduction in lever pressing, which allowed for a clearer observation of the effect of chemogenetic inhibition of CINs on slowing down this process. An intraperitoneal injection of DCZ, 30 min before each extinction session, resulted in a slower extinction learning process. The hM4Di group exhibited delayed extinction, characterized by a higher number of lever presses [Fig. 4K; group effect: *F*_(1, 15)_ = 5.02, *P* < 0.05; session effect: *F*_(1, 4)_ = 35.57, *P* < 0.001; group x session effect: *F*_(1, 60)_ = 0.79, *P* = 0.54; raw data in fig. S7B] and an increase in extinction trials, although the latter did not reach statistical significance [Fig. 4L; group effect: *F*_(1, 15)_ = 3.33, *P* = 0.09; session effect: *F*_(1, 4)_ = 29.28, *P* < 0.001; group x session effect: *F*_(1, 60)_ = 0.62, *P* = 0.65]. These findings indicate that CIN activity plays an important role in facilitating extinction learning.

### CIN firing pause is critical for reversal learning and behavioral flexibility

**Fig. 5.**
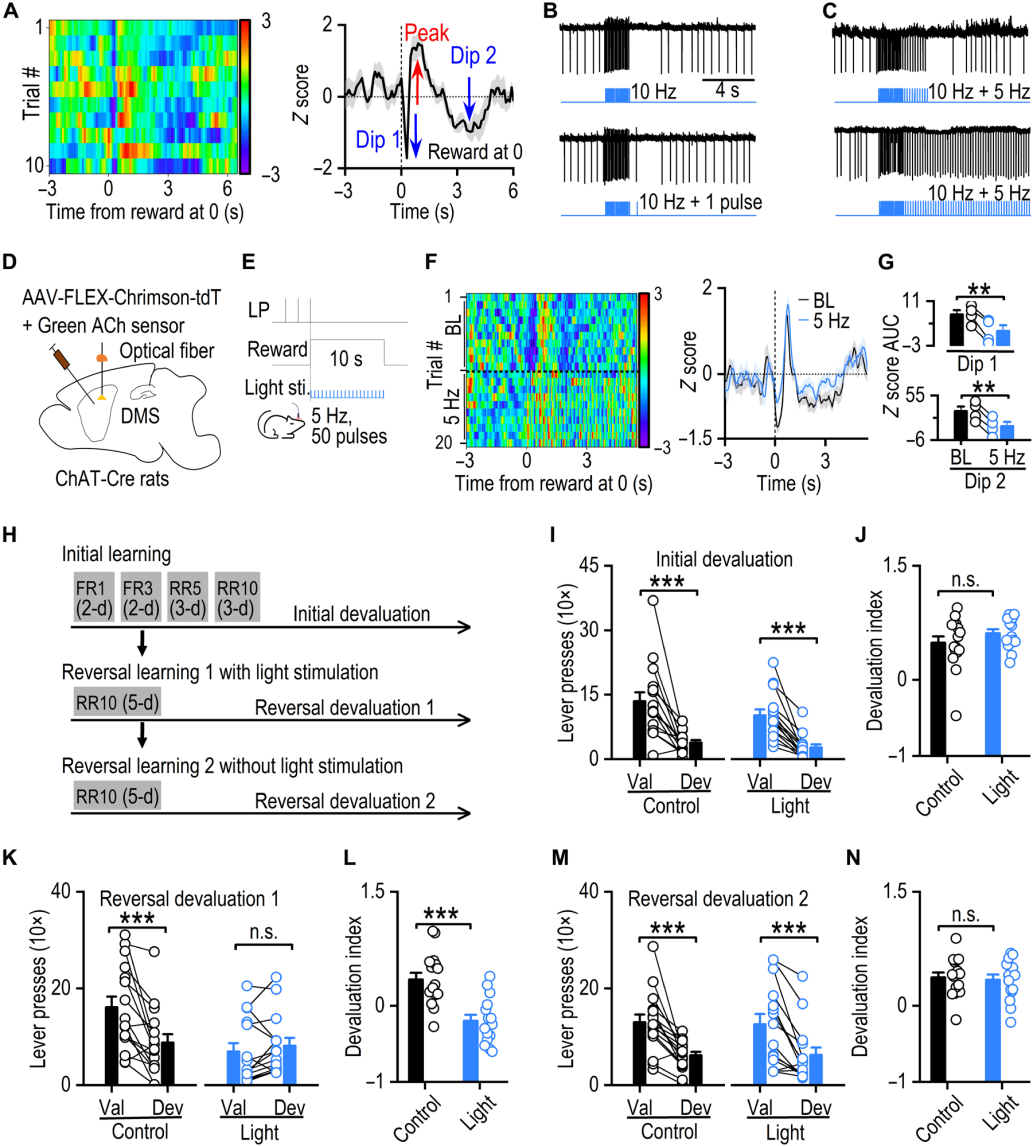
Pause in CIN firing is critical for updating goal-directed learning. (**A**) Heatmap (left) and trace (right) depict the ACh signal during training, showing a postreward decline, rebound, and subsequent drop. (**B**) Top: Optical stimulation (10 Hz, 20 pulses) induces burst-pause CIN firing in brain slices of ChAT-Cre;Ai32 mice. Bottom: An action potential can be elicited by an additional optical stimulation during the pause period. (**C**) Top: Multiple action potentials can be elicited during the pause period (5 Hz, 10 pulses). Bottom: Five-hertz continuous optical stimulation interrupts the CIN pause. (**D**) Schematic illustrating virus infusions and optical fiber implantation. (**E**) Optical stimulation protocol used during the reward delivery period. (**F**) In vivo measurements of ACh release during baseline (BL) and 5-Hz light stimulation trials, heatmap (left) and representative ACh signal traces (right). Signal traces were averaged over every five data points to reduce fluctuations [raw traces are shown in fig. S11 (F to H)]. (**G**) Summary data quantifying the dips AUC; **P < 0.01 by paired *t* test, *n* = 4 fibers from two rats. (**H**) Schematic of behavioral training. (**I**) Initial devaluation test; ***P < 0.001. (**J**) Devaluation index revealed no significant difference between groups; *P* = 0.22. (**K**) Postreversal learning devaluation test; ***P < 0.001 (control); *P* = 0.39 (light). (**L**) The devaluation index was significantly lower in the light group than in the control group; ***P < 0.001. (**M**) Postreversal learning without light stimulation devaluation test; ***P < 0.001. (**N**) There is no significant difference in the devaluation index between groups; *P* = 0.73. Mixed-model ANOVA was followed by a simple effects test for (I), (K), and (M); unpaired *t* test for (J), (L), and (N); *n* = 16 rats (control) and 15 rats (light).

After establishing the role of CIN burst firing in accelerating extinction learning, we next examined the importance of CIN pauses in behavioral flexibility, specifically during extinction and reversal learning. In our operant conditioning experiments, we observed a distinct dip-rebound-dip pattern in ACh dynamics (Fig. 5A). Since ACh dips are likely a result of CIN firing pauses, we first sought to disrupt the CIN pause ex vivo. Using DMS slices from ChAT-Cre;Ai32 mice, in which CINs express the excitatory channelrhodopin-2, we applied 10-Hz light stimulation to induce CIN bust-pause firing to mimic the ACh dynamics observed in vivo. Following this stimulation, we introduced additional light pulses—either single (Fig. 5B) or multiple (Fig. 5C)—to determine whether they could induce spikes and thereby disrupt the CIN pause. We found that 5-Hz light stimulation effectively disrupted CIN pauses ex vivo (Fig. 5C and fig. S10). Next, we tested the effect of optogenetic disruption of the CIN pause in vivo. To achieve this, we infused AAV-FLEX-Chrimson-tdT and AAV-GRAB_gACh4m_, a newer ACh sensor (*48*), together into the DMS of ChAT-Cre rats (Fig. 5D). This setup enables us to simultaneously stimulate CINs and monitor ACh signals, providing a more precise assessment of the impact of CIN pause disruption on ACh dynamics during operant conditioning. After recovery from surgery, the rats were trained to press a lever for sucrose rewards. First, we tested the effect of different frequencies of light stimulation on the ACh signal and found that higher frequencies generally induced more ACh release (fig. S11, A to C). We aimed to select a light frequency strong enough to disrupt ACh dips in vivo without inducing an additional dip afterward. Since 10-Hz stimulation induced an additional ACh dip following stimulation (fig. S11, A and B), we opted for a lower frequency, 5 Hz, to test during operant conditioning (Fig. 5E). The new ACh sensor replicated the dip-rebound-dip pattern of ACh dynamics observed with the previous sensor (e.g., Fig. 5A). Five-hertz light stimulation effectively disrupted ACh dips during operant conditioning (Fig. 5F). Quantification of the AUC showed a significant reduction of dips by 5-Hz stimulation [Fig. 5G; dip 1, *t*_(3)_ = 6.41, *P* < 0.001; dip 2, *t*_(3)_ = 16.74, *P* < 0.001], with a minimal effect on the peak phase of the ACh signal (fig. S11, D and E). These results suggest that 5-Hz light stimulation effectively disrupts CIN pauses and ACh dips.

We next examined whether disrupting CIN pauses affected the extinction process. To do this, we retrained the animals previously used in Fig. 4 (E to G) and conducted an additional extinction test, applying continuous 5-Hz optostimulation as described in Fig. 5E to specifically disrupt CIN pauses during extinction training. Our findings revealed that disrupting the CIN pauses had no discernible effect on the extinction process (fig. S12). These results suggest that CIN pauses are unlikely to play a critical role in extinction learning.

Last, we tested whether disrupting CIN pauses affects reversal learning tasks. To achieve this, we infused AAV-FLEX-Chrimson-tdT into the DMS of ChAT-Cre rats to enable optogenetic disruption of CIN pauses during reversal learning. After recovery from surgery, the rats were trained to associate the left lever with a sucrose reward and the right lever with a food reward, followed by reversal learning (Fig. 5H and fig. S3, E and F). After each learning stage, a devaluation test was conducted to assess the acquisition of these associations. Light stimulation was applied during the reversal learning phase and was time-locked to reward delivery (Fig. 5, E and H). Since it was unclear whether the initial or delayed ACh dip was critical for reversal learning, light stimulation was delivered for the entire 10 s of reward delivery to disrupt both pauses. In the initial devaluation test, both the control and light stimulation groups significantly reduced presses on the outcome-satiated devaluated lever (Fig. 5I). Statistical analysis showed a main effect of devaluation [*F*_(1,29)_ = 53.68, *P* < 0.001] with no group x devaluation interaction [*F*_(1,29)_ = 0.85, *P* = 0.36]. The devaluation index did not differ significantly between the groups [Fig. 5J; *t*_(29)_ = −1.25, *P* = 0.22]. These results indicate that both groups successfully acquired the initial action-outcome associations. However, during the reversal devaluation test, the control group maintained a preference for the valued lever, whereas the light stimulation group showed no preference, pressing both valued and devalued levers indiscriminately (Fig. 5K). Statistical analysis revealed a significant group x devaluation interaction [*F*_(1,29)_ = 16.87, *P* < 0.001]. Furthermore, simple effects analysis revealed that the control group had significantly more presses on the valued versus devalued lever [Fig. 5K, left; *F*_(1,29)_ = 25.01, *P* < 0.001], whereas the light stimulation group did not show this preference [Fig. 5K, right; *F*_(1,29)_ = 0.76, *P* = 0.39]. Notably, the light-stimulated rats had significantly lower devaluation indices than controls [Fig. 5L; *t*_(29)_ = 4.66, *P* < 0.001], suggesting that the control rats successfully acquired the reversed action-outcome associations, whereas the pause-disrupted rats did not. To test the reversibility of this disruption, we conducted further reversal learning without light stimulation. In the subsequent reversal devaluation test, the light-stimulated rats recovered their reversal learning abilities, indicating a clear preference for the valued over the devalued lever (Fig. 5M). Statistical analysis revealed a main effect of devaluation [*F*_(1,29)_ = 42.91, *P* < 0.001] with no significant group x devaluation interaction [*F*_(1,29)_ = 0.07, *P* = 0.80]. The devaluation indices were comparable between the groups [Fig. 5N; *t*_(29)_ = 0.35, *P* = 0.73]. Further analysis of devaluation indices confirmed the reversibility of light-induced disruption (fig. S13). These results suggest that CIN pauses are critical for the reversal learning process and, more broadly, for behavioral flexibility. Together, our findings indicate that although CIN pauses are unlikely to play a role in extinction learning, they are essential for successful reversal learning.

## DISCUSSION

In this study, we discovered that chronic alcohol consumption impaired thalamically evoked CIN burst-pause dynamics and reversal learning. Both reversal and extinction learning elevated ACh release in the DMS. We also found increased thalamic activity during these learning phases. The changes of action-outcome associations during extinction and reversal learning could serve as salient stimuli to stimulate thalamic neuronal activity, which could activate CINs, leading to increased ACh release. With optogenetic and chemogenetic manipulations, we showed that CIN burst firing promoted extinction and that CIN pauses were required for reversal learning. These findings demonstrate the significance of CIN burst-pause dynamics in extinction and reversal learning processes, contributing to behavioral flexibility.

### The mechanisms through which CINs modulate the extinction process

We observed dynamic responses of cholinergic signaling, including consequent ACh dip and rebound, during operant conditioning. A conceptual question arises as to how these postbehavior responses influence upcoming behavior. ACh dips may facilitate dopamine release, reinforcing subsequent reward-related actions (*49*), while ACh rebounds might inhibit medium spiny neurons (MSNs) involved in reward-taking (*47*, *50*, *51*), aiding the transition between reward-seeking and reward-taking behaviors (*52*). Research has indicated that CIN activity and ACh elevation are associated with the extinction of cocaine memories and Pavlovian conditioning (*53*–*55*). Our findings suggest that CIN burst firing facilitates the extinction of operant conditioning. This modulation is likely through ACh influencing downstream targets. The striatum has the highest activity of acetylcholinesterase (AChE; the enzyme degrading acetylcholine) in the brain (*14*, *17*, *56*), indicating tight control over ACh levels. As the primary ACh source in the striatum (*17*), CIN burst firing can elevate striatal ACh levels. ACh can have distinct modulations on different striatal cell types through different receptors. For example, via M4 muscarinic receptors (M4Rs), ACh can suppress excitatory inputs to dopamine D1 receptor–expressing medium spiny neurons (D1-MSNs) (*13*, *57*), promoting long-term depression in these neurons (*57*). This could help reduce lever presses during extinction when ACh no longer increases. Notably, M4Rs are known to negatively regulate D1-MSN function (*58*). In addition, through M1 muscarinic receptors, ACh can heighten short-term excitability in dopamine D2 receptor–expressing MSNs (D2-MSNs) (*13*, *28*). Considering that striatal D2-MSNs lead to the “No-Go” pathway, this indicates that CIN activation might aid extinction by enhancing the No-Go action. This aligns with research showing enhanced D2-MSN activation during extinction training (*59*, *60*). Another mechanism through which ACh could promote extinction involves activating GABAergic interneurons in the striatum through nicotinic acetylcholine receptor (nAChR) (*61*, *62*). The activation of these GABAergic interneurons could exert robust inhibition over striatal MSN activity, and our recent study shows that D1-MSN inhibition during the reward delivery period facilitates extinction (*51*).

Our burst optogenetic activation of CINs is often followed by a pause, so the question remains whether the burst or pause mediates the behavioral effects of our manipulation. When we disrupted the pauses during extinction learning, we found no effect on the extinction process. This suggests that the CIN pause is unlikely to play a role in extinction learning. The experiment with optogenetic inhibition of CINs also provides some insights as we can consider opto-inhibition as a long pause. When we introduced this long pause during extinction training, there was no effect overall. On the basis of the above evidence, we believe that, in our studies with optogenetic CIN activation, the induced burst firing rather than the pause mediates the enhanced extinction behavioral effect.

It is generally believed that burst firing of CINs corresponds to increased ACh release. However, it is important to acknowledge the potential dissociation between CIN somatic firing and local ACh release. Similar to the nonlinear relationship observed between dopamine neuron firing and striatal dopamine release (*63*), several mechanisms can modulate ACh release independent of somatic firing. These include terminal modulation via G protein–coupled receptors, such as dopamine D2 receptors and muscarinic ACh receptors (*64*). For example, dopamine can inhibit ACh release via D2 receptors on CINs (*25*), while ACh can exert negative feedback through M2/M4 autoreceptors (*65*). In addition, purines and other regulatory signals also play a role in modulating ACh release (*66*). AChE is another key factor that degrades ACh and regulates local ACh levels. AChE is differentially distributed across the striatum, which further contributes to variations in local ACh regulation (*67*, *68*).

### The mechanisms of CIN pause and its behavioral application

The behavioral function of CIN pauses is still unclear. Multiple mechanisms have been proposed for pause formation in CINs, including inhibitory γ-aminobutyric acid (GABA) synaptic inputs (*47*, *61*) and intrinsic properties to generate membrane potential hyperpolarization following excitation (*69*, *70*). Dopamine signals have been implicated in the regulation of the pause response of CINs, with a particular interest in dopamine D2 receptor–mediated effects (*25*, *71*). The D2 receptor is expressed in CINs (*72*), and dopamine has been found to regulate multiple intrinsic conductances of CINs through this receptor (*73*, *74*). This regulation of CINs has been shown to be a key mediator of dopamine-dependent striatal plasticity (*75*). Our study demonstrates that a pause in CIN firing is critical for the reversal of goal-directed learning. CIN tonic firing maintains a consistent ACh baseline level in the striatum. This ACh generally serves as an inhibitory signal by suppressing corticostriatal transmission via presynaptic M2 receptors (*28*) and inhibiting MSNs through GABAergic interneurons (*47*, *61*, *62*, *76*). When CINs pause their firing, ACh release stops and is quickly cleared because of the rapid catabolic activity of AChE (*77*). This ACh-free period, combined with dopamine signaling, is crucial for striatal-dependent learning (*18*, *23*, *24*, *32*). A recent study showed that, for long-term corticostriatal plasticity to occur, the increase in dopamine, the CIN pause, and MSN depolarization must synchronize (*33*). This suggests that the pause in CIN firing is critical for neuronal plasticity to occur and thus could play an essential role in changing animal behavior. Disrupting this pause showed that it is vital for reversal learning, which is consistent with the finding that reducing the CIN pause duration by chronic alcohol consumption impairs reversal learning (*13*). For successful reversal learning, animals need to extinguish prior action-outcome associations and update their behavior with new ones. The question then arises as to what aspect of reversal learning is affected by our pause-disruption manipulation. We reason that our pause-disruption manipulation impaired reversal learning due to a failure to update new action-outcome associations but not a failure to extinguish the prior associations. There are several lines of evidence supporting this conclusion. First, our data showed that disrupting the pause did not affect the extinction process. Second, our 5-Hz optical stimulation caused a slight increase in the ACh peak, which has been shown to promote extinction. Third, if the rats failed to extinguish prior associations, then they would still show some preference for the previously valued lever (the devalued lever after reversal learning), which was observed in alcohol-drinking rats but not in pause-disrupted rats. Therefore, our data show that the CIN pause is critical for updating goal-directed learning.

### The synergy of CIN burst and pause for promoting cognitive flexibility

Through experimental manipulations, we conclude that the burst firing of CINs is important for extinction, while pauses in CIN firing are vital for updating learning. In the natural environment, the burst and pause patterns of CIN firing may work together to promote cognitive flexibility: Burst firing aids in old learning extinction, while the subsequent pause assists with new learning. The impaired reversal learning in alcohol-exposed animals may stem from both reduced burst and pause responses. The former hinders the extinction of previous learning, while the latter affects the updating of new learning. During the devaluation test after reversal learning, the alcohol group overall still had a higher number of presses on the devalued lever (the previously valued lever) than the valued lever, indicating that the previous action-outcome associations had not been fully extinguished. As for alcohol’s impact on CIN responses, our previous study indicated that its effect is unlikely to be due to changes in presynaptic release because of the unaffected paired-pulse ratio (*13*). We suspect instead that postsynaptic changes in AMPA receptor functions play a role, as evidenced by decreased AMPA-induced currents in alcohol-exposed CINs. Another possible factor affecting thalamically induced burst-pause responses is changes in CIN baseline firing frequency. We discovered that chronic alcohol intake and withdrawal increased the spontaneous firing of CINs. Optimal ACh signaling is vital for attention and learning (*78*), and there appears to be an ideal baseline CIN activity in the striatum for modulating its dynamic responses. Any deviation from this optimal baseline may compromise the adaptability of CINs to salient behavioral cues.

### The multiphasic stages of CIN dynamic response during behavior

CINs display very dynamic responses during behaviors (*26*, *27*). During our instrumental learning paradigm, we noted CINs exhibiting multiphasic responses. Typically, during reward delivery, the ACh signal dips, surges, and then dips again. What causes these changes in ACh signals are unclear. On the basis of previous literature, we believe that the initial dip might result from GABA-mediated CIN inhibition (*26*, *27*), as CINs receive multiple inhibitory GABAergic inputs, including those from striatal MSNs (*79*–*81*), and can self-inhibit by activation of GABAergic interneurons through nAChR (*47*, *61*). For the initial dip, no preceding CIN excitation (excitation could initiate CIN self-inhibition) was seen. We hypothesize that this dip is triggered by GABA release from D1-MSNs during reward-acquiring actions, with supporting evidence from our observation that D1-MSN optogenetic activation can induce a pause-rebound in CIN firing (*82*). The subsequent ACh increase may have dual origins: pause-rebound firing of CINs (*26*, *82*) and thalamic CIN activation (*28*, *31*). The delayed ACh dip might arise from another CIN firing pause, often seen in postthalamic activation (*28*, *83*). Self-initiated recurrent inhibition of CINs (*47*, *61*) could also play a role. Moreover, CIN pause response could be modulated by dopamine signals (*28*, *84*–*86*). Although the mechanisms for the multiphasic responses of CINs are not entirely clear, this pause and rebound, followed by a second pause of CINs, has been repeatedly observed during animal behavior (*87*–*89*). One limitation of this study is that the temporal resolution of our pause-disruption manipulation is not high, with light stimulation throughout the reward delivery period covering the initial dip, middle rise, and late decrease. Future studies are needed to elucidate the mechanisms of CIN dynamic responses, dissect out which pause response is critical for updating goal-directed learning, and explore how various signals collaborate during the pause to enhance neural plasticity in behavior.

In summary, our study discovered that thalamic-driven CIN burst firing promotes extinction learning, while the pause in CIN firing is pivotal for reversal learning of goal-directed behavior. A deeper understanding of the behavioral implications of CIN dynamic responses will pave the way to elucidate their critical functions in both the healthy and diseased brains.

## MATERIALS AND METHODS

### Animals

ChAT-eGFP (stock #007902), VGluT2-Cre (stock #016963), and Ai32 (stock #012569) mice were purchased from the Jackson Laboratory. All mice were backcrossed onto a C57BL/6J background. VGluT2-Cre mice were crossed with Ai32 to generate the VGluT2-Cre;Ai32 line. VGluT2-Cre;Ai32 mice were crossed with ChAT-eGFP to generate VGluT2-Cre;Ai32;ChAT-eGFP triple transgenic mice. Both male and female mice were used for electrophysiology studies. Long-Evans rats (3 months old) were purchased from Harlan Laboratories. LE-Tg(ChAT-Cre)5.1Deis rats were purchased from Rat Resource & Research Center (stock #00658). ChAT-Cre rats were bred in-house. Both male and female rats (3 months or older) were used for behavioral testing. Rats were used in behavioral experiments because they are easier to handle and are less stressed by handling. In addition, they usually perform better than mice in operant learning tasks. For electrophysiological recordings, diverse transgenic mouse lines were used for cell type–specific measurements, such as VGluT2-Cre;Ai32;ChAT-eGFP mice, which are now unavailable in rats. Animals were housed individually at 23°C under a 12-hour light:dark cycle, with lights on at 7:00 a.m. Food and water were provided ad libitum. All animal care and experimental procedures were approved by the Texas A&M University Institutional Animal Care and Use Committee (2022-0198) and were conducted in agreement with the National Research Council Guide for the Care and Use of Laboratory Animals.

### Reagents

D-(-)-2-Amino-5-phosphonopentanoic acid (catalog #ab120003 [#ab12003]) was purchased from Abcam. DNQX (6,7-dinitroquinoxaline-2,3-dione) (#0189) was purchased from Tocris. DCZ and other chemicals for electrophysiological recordings were obtained from Sigma-Aldrich. DCZ was dissolved in 2% dimethyl sulfoxide in saline. For behavioral experiments, DCZ was intraperitoneally injected at 0.2 mg/kg; for bath application, it was diluted to 1 μM. rAAV8/Syn-Chrimson-tdT (#AV5841D), rAAV8/Syn-FLEX-Chrimson-tdT (#AV5844D), rAAV8/Syn-Chronos-GFP (#AV5842B), rAAV8/Syn-DIO-hM4Di-mCherry (#AV4980F), and rAAV8/Syn-hM4Di-mCherry (#AV5360C) were purchased from the University of North Carolina Vector Core. pAAV.CAG.iAChSnFR (#137955-AAV9) and pAAV.Syn.GCaMP6s.WPRE.SV40 (#100843-AAV5) were purchased from Addgene. rAAV/hSyn-GRAB_gACh4m_ (#PT-7021) was purchased from BrainVTA.

### Behavioral procedures

#### Intermittent access to 20% alcohol two-bottle choice drinking procedure

This procedure was conducted as described previously (*36*, *37*, *90*–*96*). Briefly, animals were given concurrent access to one bottle of alcohol (20%, in water) and one bottle of water for 24-hour periods, which were separated by 24- or 48-hours periods of alcohol deprivation. Alcohol intake (grams per kilogram per day) was calculated by determining the weight of 20% alcohol solution consumed and multiplying this by 0.2. Water control animals only had access to water.

#### Operant conditioning training

Operant conditioning was conducted on rats as previously described (*13*, *82*, *92*, *97*). Blinding was applied to behavioral experiments. An independent observer coded and randomized animals using a computer-generated blinding algorithm. Researchers in the lab trained rats without knowing the treatment plan for the animals. Food was restricted to maintain 80% of the original body weight of the animals for the duration of behavioral studies.

#### Magazine training

This procedure was adapted from previous studies (*7*, *13*). After 5 days of food restriction, rats were trained for magazine entries for two consecutive days. During these training sessions, a reinforcer (either a food pellet or 0.1 ml of sucrose solution) was delivered along with illumination of the magazine light for 1 s with a random interval between each reinforcer (on average of 60 s). For each day, rats received either 20 food pellets or 20 sucrose deliveries during the first training session and were then switched to receive the other reward in the second training session. The house light was illuminated throughout the session, and no levers were available during magazine training.

#### Acquisition of initial contingencies

Following magazine training, rats were trained to access different reinforcers via lever pressing over sessions. The lever was retracted during the reinforcer’s delivery. Each session consisted of four blocks (two blocks per lever), separated by a 2.5-min timeout during which no levers were available, and all lights were extinguished. Only one lever was available during each block (pseudorandom presentation), which lasted for 10 min or until 10 reinforcers had been earned. For extinction sessions, only two blocks (one block per lever, 5 min for each block), separated by a 1.5-min timeout, were used. For half of the animals in each group, the left lever was associated with food pellet delivery and the right lever with sucrose solution delivery. The remaining animals were trained using the opposite pairs of action-outcome associations. Lever training started with a fixed ratio 1 (FR1) schedule where every lever press resulted in the delivery of a reinforcer. Some animals needed more training sessions to form the association between lever pressing and reward delivery. After 2 days of stable FR1 training, the training schedule was elevated to FR3 for 2 days. Then, we proceeded to a random ratio 5 (RR5) schedule for the next 3 days, during which a reinforcer was delivered after lever pressing with a probability of 0.2. An RR10 (or a probability of 0.1) training schedule was then used for 3 days. For chemogenetic behavioral experiments, rats were trained in a one-block 30-min session to press a lever for a total of 40 sucrose rewards from FR1, FR3, and RR5 to RR10; each extinction session was 30 min and used the RR5 protocol. A single reward (sucrose) was used consistently across all chemogenetic experiments (Figs. 3, K to O, and 4, H to L, and figs. S3D, S6, and S7B) and in one optogenetic study that disrupted ACh dips (Fig. 5, D to G, and fig. S11).

#### Devaluation test

After the final RR10 training, devaluation testing was performed for 2 days. On both days, rats were habituated in a dark, quiet room (different from the operant room) for 30 min and then were given ad libitum access to either the food pellets (25 g placed in a bowl) or the sucrose solution (100 ml in a drinking bottle) in a devaluation cage for 1 hour. The devaluation cage was similar to their home cage but with new bedding. The rats were then placed in the operant chamber for a 10-min extinction choice test. Both levers were extended during this test, but no outcomes were delivered in response to any lever press. On the second devaluation day, the rats were prefed, as described, with the other reward before repeating the same extinction test. If rats fail to perform during the devaluation test, then the prefeed reward amount and duration need to be tailored to individual animals. Lever presses were recorded, and those on the lever that the rat had learned to associate with the nondevalued reward were termed Val_Lever_, while those on the lever associated with the devalued reward were termed Dev_Lever_. The devaluation index [(Val_Lever_ − Dev_Lever_)/(Val_Lever_ + Dev_Lever_)] was then used to determine the extent of goal-directed versus habitual behavior.

#### Contingency reversal and devaluation testing

After the devaluation test, rats were retrained on their current action-outcome associations for 1 day. The contingencies were then reversed so the lever that previously delivered food now delivered sucrose, and the rats were trained using the RR10 schedule. All other procedures were unchanged. The contingency reversal training lasted for 5 days. The rats then underwent devaluation testing again using the procedure described above.

### Electrophysiology

Slice electrophysiology was performed as previously described (*91*, *96*, *98*, *99*). Animals were weaned around postnatal 21 days and consumed 20% alcohol for 6 to 8 weeks in the intermittent-access two-bottle choice drinking procedure. Animals were euthanized 1 day or 21 days after their last period of alcohol consumption, and 250-μm coronal sections containing the striatum were prepared in an ice-cold cutting solution containing 40 mM NaCl, 148.5 mM sucrose, 4 mM KCl, 1.25 mM NaH_2_PO_4_, 25 mM NaHCO_3_, 0.5 mM CaCl_2_, 7 mM MgCl_2_, 10 mM glucose, 1 mM sodium ascorbate, 3 mM sodium pyruvate, and 3 mM myoinositol, saturated with 95% O_2_ and 5% CO_2_. Slices were then incubated in a 1:1 mixture of cutting solution and external solution at 32°C for 45 min. The external solution contained the following 125 mM NaCl, 4.5 mM KCl, 2.5 mM CaCl_2_, 1.3 mM MgCl_2_, 1.25 mM NaH_2_PO_4_, 25 mM NaHCO_3_, 15 mM sucrose, and 15 mM glucose, saturated with 95% O_2_ and 5% CO_2_. Slices were then maintained in an external solution at room temperature until use.

Slices were perfused with the external solution at a flow rate of 3 to 4 ml/min at 32°C. The CINs in the DMS were identified either by differential interference contrast or by fluorescence. Whole-cell patch-clamp and cell-attached recordings were made using a MultiClamp 700B amplifier controlled by pClamp 10.4 software (Molecular Devices). Optogenetically evoked CIN firing was induced by light stimulation through the objective lens. Chronos was activated by a 470-nm laser; Chrimson was activated by a 590-nm laser. For cell-attached and whole-cell current-clamp recordings, we used a K^+^-based intracellular solution containing 123 mM potassium gluconate, 10 mM Hepes, 0.2 mM EGTA, 8 mM NaCl, 2 mM MgATP, and 0.3 mM NaGTP (pH 7.3), with an osmolarity of 270 to 280 mOsm. Data were analyzed using Clampfit (in pClamp 10.7, Molecular Devices).

### In vivo fiber photometry recording

Fiber photometry was conducted as previously described (*82*, *100*). Rats were connected to the fiber before data collection to acclimate to the fiber patch cable. During the data collection session, the spectrum data were recorded continuously at 10 Hz using OceanView 1.6.7. At the same time, behavior data were collected. A 488-nm laser was delivered to excite iAChSnFR or GCaMP6s. For the simultaneous measuring of GRAB_gACh4m_ and optical stimulation of Chrimson, the fiber photometry machine FP3002 was used (Neurophotometrics, San Diego). The percentage Δ*F/F* was calculated by 100 × (*F* − *F*_mean_)/*F*_mean_, where *F*_mean_ was the mean fluorescence intensity. The *Z* score (*Z*) was calculated using MATLAB by

$$Z=\frac{\Delta F/F-\mu }{\sigma }$$

To analyze the fiber photometry data in the context of rat behavior, MATLAB scripts were developed. The code used for the analysis of fiber photometry data is available online at the Zenodo public repository (https://doi.org/10.5281/zenodo.7948766). To normalize signals across animals and sessions, we calculated a single-baseline fluorescence value for each trial using the average of the first 5 s of 10-s period before the reward delivery and subtracted that from the signal. Peak amplitudes were calculated by taking the maximum value between 1 s before and 2 s after the reward delivery. The AUC values were restricted to time windows 0 to 5 s after the reward delivery.

### Stereotaxic surgery and histology

Stereotaxic viral infusions were performed as described previously (*36*, *98*, *101*). Briefly, animals were anesthetized using isoflurane and mounted in a rodent stereotaxic frame (Kopf). The skin was opened to uncover the skull and expose Bregma and Lambda and the location of the desired injection site. A three-axis micromanipulator was used to measure the spatial coordinates for Bregma and Lambda. Small drill holes were made in the skull at the appropriate coordinates, according to the Paxinos atlas (*102*, *103*). Two microinjectors were loaded with the virus and then lowered into the appropriate coordinates. The virus was infused into the brain at a rate of 0.1 μl/min. To avoid backflow of the virus, microinjectors were left in place for 10 min after the infusion was complete. Following their removal, the skin was sutured, and the animals were allowed to recover for at least 1 week before the next experiment. For fiber implantation, following virus injections, bilateral optical fiber implants (300-μm core fiber secured to a 1.25-cm ceramic ferrule with 5 mm of fiber extending past the end of the ferrule) were lowered 0.1 mm above the virus injection sites. Implants were secured on the skull using metal screws and dental cement (Henry Schein) and covered with denture acrylic (Lang Dental). The incision was closed around the head cap, and the skin was vet-bonded to the head cap. The coordinates for mice are as follows: PfN (anterior-posterior (AP), −2.2; medial-lateral (ML), ±0.7; dorsal-ventral (DV), −3.5 mm) for AAV-Chronos-GFP (0.5 μl) and AAV-FLEX-Chrimson-tdT (0.5 μl). The coordinates for rats are as follows: DMS (AP, 0.0; ML, ±2.8; DV, −4.85 mm) for AAV-FLEX-Chrimson-tdT(1 μl), AAV-iAChSnFR (1 μl), AAV-DIO-hM4Di-mCherry (1 μl), and AAV-FLEX-Chrimson-tdT + AAV-GRAB_gACh4m_ (2:1 mix, 1 μl); PfN (AP, −4.2; ML, ±1.25; DV, −6.2 mm) for AAV-GCaMP6s (1 μl) and AAV-hM4Di-mCherry (1 μl).

The histology procedure was performed as described previously (*98*, *104*). Briefly, mice were anesthetized and perfused intracardially with 4% paraformaldehyde in phosphate-buffered saline (PBS). Whole brains were taken out and placed into 4% paraformaldehyde in PBS for postfixation overnight (4°C) and then moved to 30% sucrose in PBS (4°C) and allowed to sink to the bottom of the container before preparing for sectioning. Frozen brains were cut into 50-μm coronal sections on a cryostat. A confocal laser-scanning microscope (Fluorview-1200, Olympus) was used to image these sections with a 470-nm laser (to excite enhanced yellow fluorescent protein and green fluorescent protein) and a 593-nm laser (to excite tdT). All images were processed using Imaris 8.3.1 (Bitplane, Zurich, Switzerland).

### Statistical analysis

All data are expressed as mean ± SEM. Statistical analyses were performed using SigmaPlot 12.0 (Systat Software Inc.). Normal distribution was tested; unpaired *t* test, paired *t* test, and one-way analysis of variance (ANOVA) or two-way ANOVA with repeated measures followed by Tukey post hoc test were used to determine statistical significance as appropriate, with an α value of 0.05. Mixed-model ANOVA followed by a simple effects test was conducted for Figs. 1 (L and N) and 5 (I, K, and M) in SPSS 29.0 (*12*).
