# Supplementary material for: Dynamic responses of striatal cholinergic interneurons control behavioral flexibility
Source: Sci Adv. 2024 Dec 18;10(51):eadn2446. doi: 10.1126/sciadv.adn2446 (PMC11654678; doi:10.1126/sciadv.adn2446)
Supplement: Supplementary file 1 — Figs. S1 to S13 [file sciadv.adn2446_sm.pdf]

Science Advances
AAAS

Supplementary Materials for

Dynamic responses of striatal cholinergic interneurons control behavioral flexibility

Zhenbo Huang *et al.*

Corresponding author: Jun Wang, jwang188@tamu.edu

Sci. Adv. **10**, eadn2446 (2024)
DOI: 10.1126/sciadv.adn2446

This PDF file includes:

Figs. S1 to S13

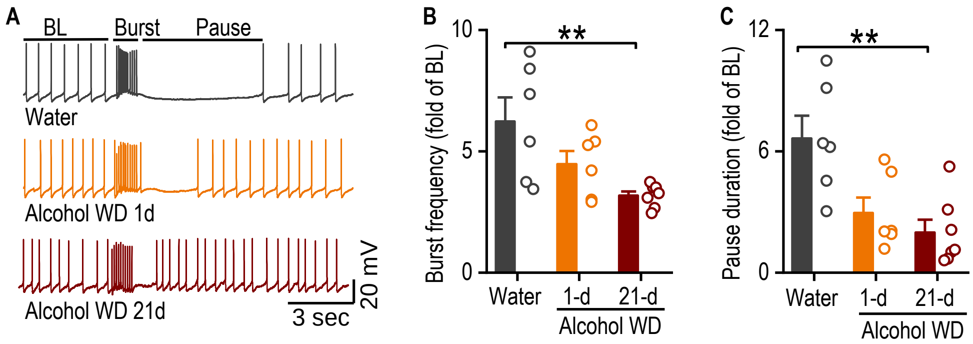

Fig. S1. Whole-cell recording of CINs from Figure 1B.
(A) Sample traces from whole-cell recording of CINs in response to optical stimulation of thalamic inputs (470 nm, 10 Hz, 10 pulses). (**B**) Bar graph of burst frequency expressed as fold change of baseline firing frequency. $F_{(2,16)} = 6.25$, $^{**}p < 0.01$. (**C**) Bar graph of pause duration expressed as fold change of baseline interspike intervals. $F_{(2,16)} = 8.29$, $^{**}p < 0.01$. One-way ANOVA followed by Tukey *post-hoc* test, n = 6 neurons from 3 mice (6/3) for Water, 6/3 for Alcohol WD 1-d, and 7/3 Alcohol WD 21-d groups.

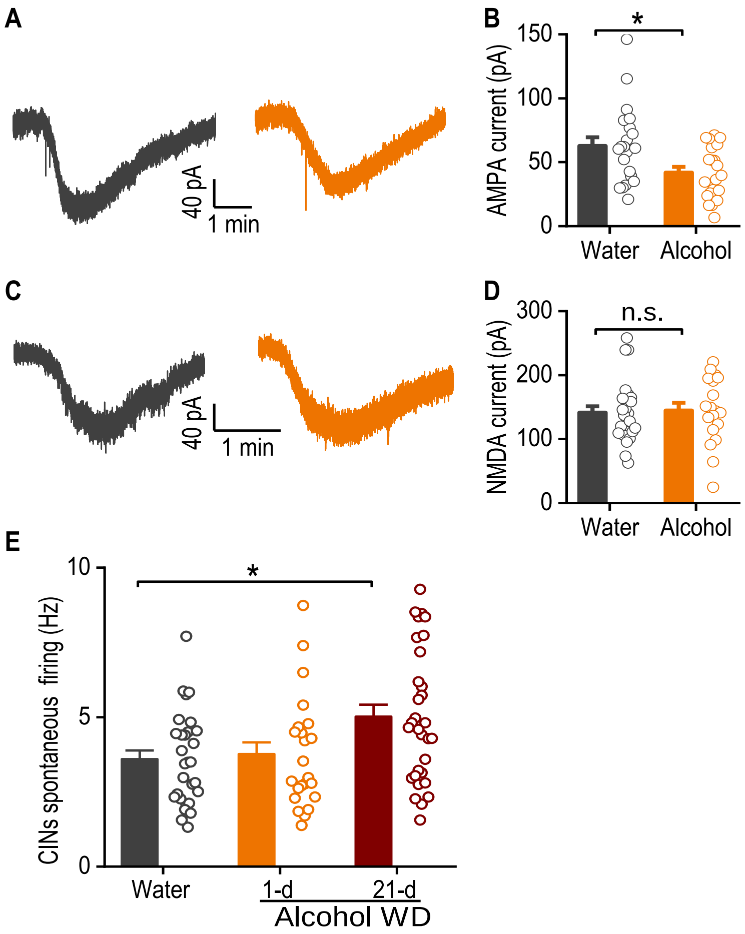

Fig. S2. Chronic alcohol intake reduced AMPA-induced currents in DMS CINs.
(**A**) Sample traces of bath application of AMPA-induced (10 µM, 15 seconds) currents of CIN in water (left) and alcohol (right) drinking animals. (**B**) Summary data quantifying the peak currents. Unpaired *t* test, $t_{(40)} = 2.62$, $*p < 0.05$, n = 21 neurons from 5 mice (Water 21/5) and (Alcohol 21/4). (**C**) Sample traces of bath application of NMDA (30 µM, 30 seconds) induced currents of CIN in water (left) and alcohol (right) drinking animals. NMDA-induced currents were recorded in a magnesium-free external solution. (**D**) Summary data quantifying the peak currents. Unpaired *t* test, $t_{(41)} = -0.23$, $p = 0.82$, n = (Water 24/5) and (Alcohol 19/4). (**E**) Spontaneous firing rates of CINs in the indicated groups; one-way ANOVA $F_{(2,80)} = 4.79$, $p = 0.01$, $*p < 0.05$ vs. water group by Tukey *post hoc* test; n = (Water 29/5), (Alcohol WD1-d 23/5), and (Alcohol WD 21-d 31/5).

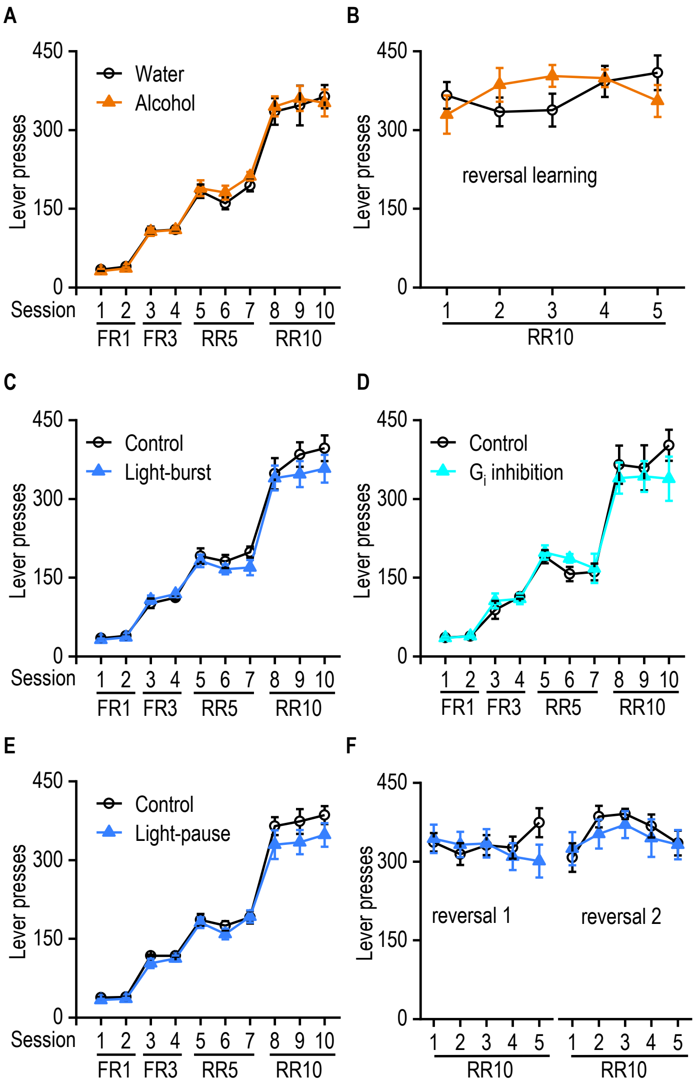

Fig. S3. Learning curves of operant conditioning.

Rats were trained in the operant chambers to press a lever for rewards, progressing from fixed ratio (FR) protocols FR1 and FR3 to random ratio (RR) protocols RR5 and RR10. Reversal learning used the RR10 protocol. (**A**) The initial learning curve of animals as in Figure 1. There was a main effect of session ($F_{(1,9)} = 144.96$, $p < 0.001$) but no group effect ($F_{(1,19)} = 0.14$, $p = 0.71$). (**B**) The reversal learning curve of animals as in Figure 1. There was no group effect ($F_{(1,19)} = 0.27$, $p = 0.61$). n = 11 rats (Water) and 10 rats (Alcohol). (**C**) The learning curve of animals as in Figure 4F. There was a main effect of session ($F_{(1,9)} = 156.99$, $p < 0.001$) but no group effect ($F_{(1,24)} = 1.45$, $p = 0.24$). n = 13 rats (Control) and 13 rats (Light-burst). (**D**) The learning curve of animals as in Figure 4K. There was a main effect of session ($F_{(1,9)} = 86.01$, $p < 0.001$) but no group effect ($F_{(1,15)} = 0.22$, $p = 0.65$). n = 9 rats (Control) and 8 rats (Gi-inhibition). (**E**) The initial learning curve of animals as in Figure 5H. There was a main effect of session ($F_{(1,9)} = 217.29$, $p < 0.001$) but no group effect ($F_{(1,29)} = 2.40$, $p = 0.13$). (**F**) The reverse learning curve of animals as in Figure 5H. There was no group effect ($F_{(1,29)} = 0.25$, $p = 0.62$). n = 16 rats (Control) and 15 rats (Light-pause).

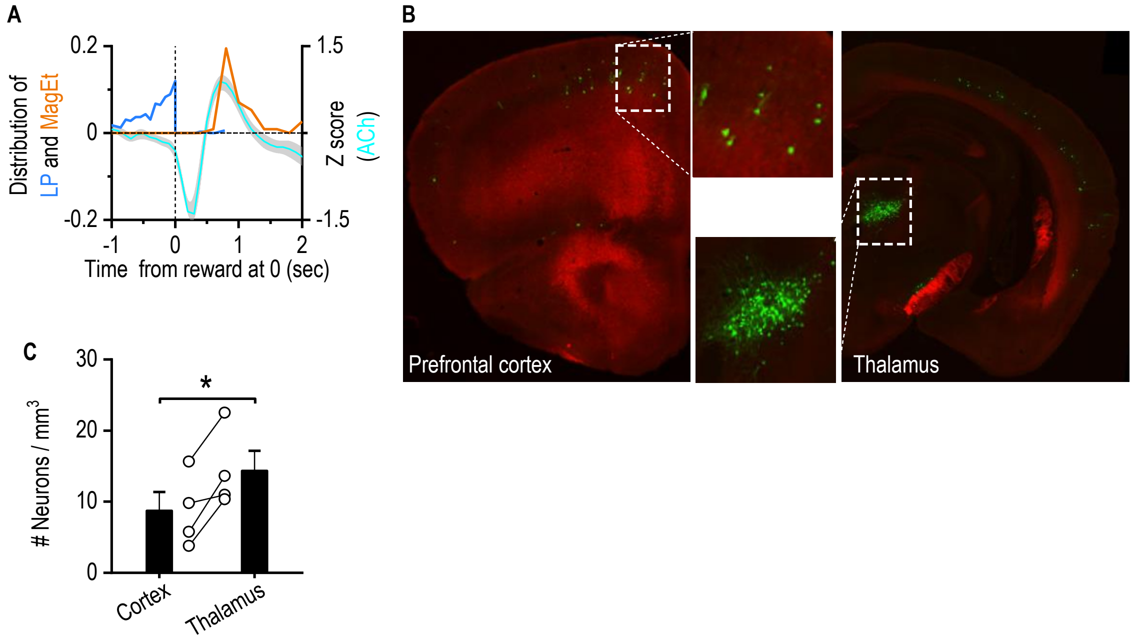

Fig. S4. Rabies virus tracing of input neurons to striatum CINs.
(A) Overlay the behavioral distribution graph in Figure 2G with the ACh signal trace of initial learning in Figure 2I. For the rabies virus tracing experiment, Cre-dependent helper viruses (AAV-DIO-TVA (EnvA receptor)-mCherry and AAV-DIO-RG (rabies glycoprotein) were infused into the DMS of ChAT-Cre;D1-tdTomato mice, followed by rabies virus (RV-GFP) infusion at the same site 3 weeks later. Coronal sections were prepared 1 week after rabies virus infusion. (**B**) Representative coronal sections of the prefrontal cortex and thalamus showing GFP-positive input neurons. (**C**) Summary data of input neuron density comparing cortex and thalamus, paired *t* test, $t_{(3)} = -3.73$, $*p < 0.05$, n = 4 mice.

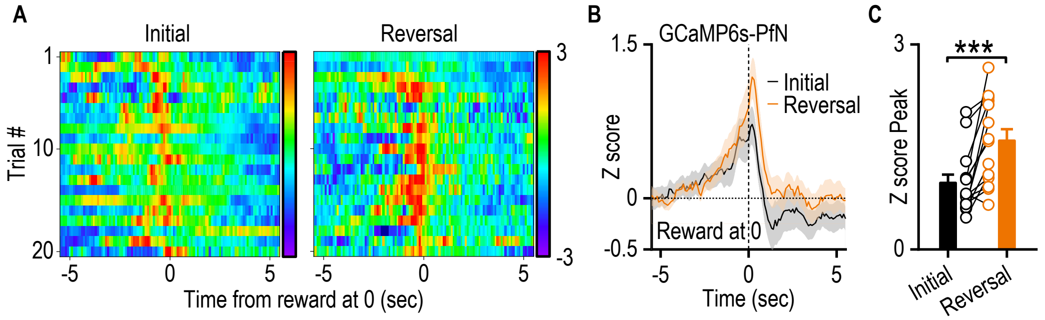

Fig. S5. Enhanced PfN activity during reversal learning.
(**A**) *In vivo* measurements of GCaMP signals in the PfN. The heat map showed GCaMP signals during the first reversal session, which consisted of initial learning in the first half session and reversal learning in the second half. (**B**) Representative traces of GCaMP signals during the first reversal session. (**C**) Summary data quantifying the peak GCaMP sensor fluorescence signals, $t_{(13)} = -4.41$, $***p < 0.001$ by paired t test. n = 7 sucrose reversal sessions + 7 food reversal sessions from 7 rats.

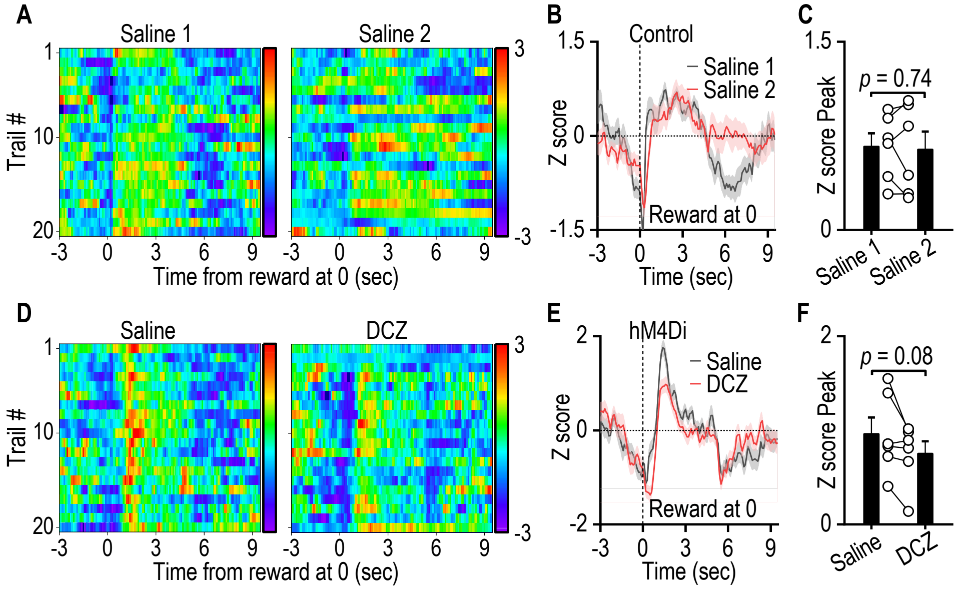

Fig. S6. Analysis of ACh release in reinforced sessions across days.
(**A**) *In vivo* measurements of ACh signals in the DMS across days in the control group. Saline was injected on different days. (**B**) Representative traces of ACh signals across days in the control group. (**C**) Summary data quantifying the peak ACh sensor fluorescence signals; paired *t* test, $t_{(5)} = 0.35$, $p = 0.74$. (**D**) *In vivo* measurements of ACh signals in the DMS across days in the hM4Di group. Saline was injected one day, while deschloroclozapine (DCZ) was injected another day. (**E**) Representative traces of ACh signals across days in the hM4Di group. (**F**) Summary data quantifying the peak ACh sensor fluorescence signals; paired *t* test, $t_{(5)} = 2.15$, $p = 0.08$. n = 6 rats (Control), n = 6 rats (hM4Di).

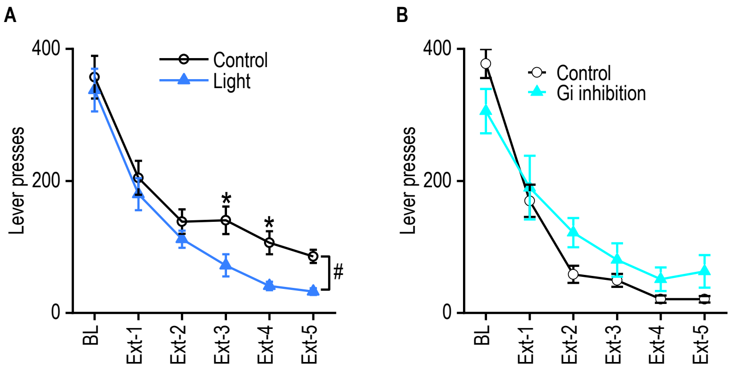

Fig. S7. Raw lever press data.

(**A**) Raw lever press data for Figure 4F. Group effect: $F_{(1, 24)} = 7.68$, $p < 0.05$; session effect: $F_{(1, 4)} = 30.19$, $p < 0.001$; group x session effect: $F_{(1, 96)} = 1.20$, $p = 0.32$. Two-way RM ANOVA followed by Tukey *post-hoc* test, $^{\#}p < 0.05$, $^{*}p < 0.05$, n = 13 rats (Control) and 13 rats (Light).

(**B**) Raw lever press data for Figure 4K. Group effect: $F_{(1, 15)} = 2.11$, $p = 0.17$; session effect: $F_{(1, 4)} = 35.95$, $p < 0.001$; group x session effect: $F_{(1, 60)} = 0.67$, $p = 0.62$.Two-way RM ANOVA followed by Tukey *post-hoc* test, n = 9 rats (Control) and 8 rats (G_i-inhibition).

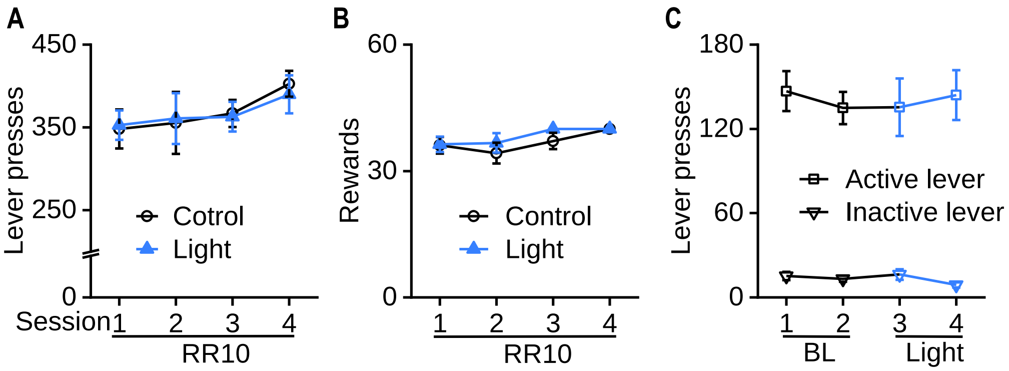

Fig. S8. Optical stimulation of CINs while rewards are available.
(**A**) Rats were trained in the operant chambers to press a lever for rewards (sucrose and food) using the RR10 protocol. Lever presses triggered reward and synchronized light stimulation (590 nm) time-locked to reward delivery. Light stimulation comprised 5 repetitions of 20 Hz light bursts (10 pulses, 5 ms per pulse) with a 0.8-second interval. There was no group effect ($F_{(1,16)}$ = 0.007, p = 0.94). (**B**) The earned rewards during training were not significantly different between the two groups ($F_{(1,16)}$ = 0.90, p = 0.36). n = 9 rats (Control) and 9 rats (Light). (**C**) Rats were trained in the operant chambers to press the active lever for a reward (20% alcohol) using the FR3 protocol. Once the animals reached two stable baseline (BL) session performances, light stimulation (590 nm) time-locked to reward delivery was administrated for the next two sessions. Light stimulation comprised 5 repetitions of 20 Hz light bursts (10 pulses, 5 ms per pulse) with a 0.8-second interval. There was no session effect ($F_{(1,3)}$ = 0.23, p = 0.88). n = 7 rats.

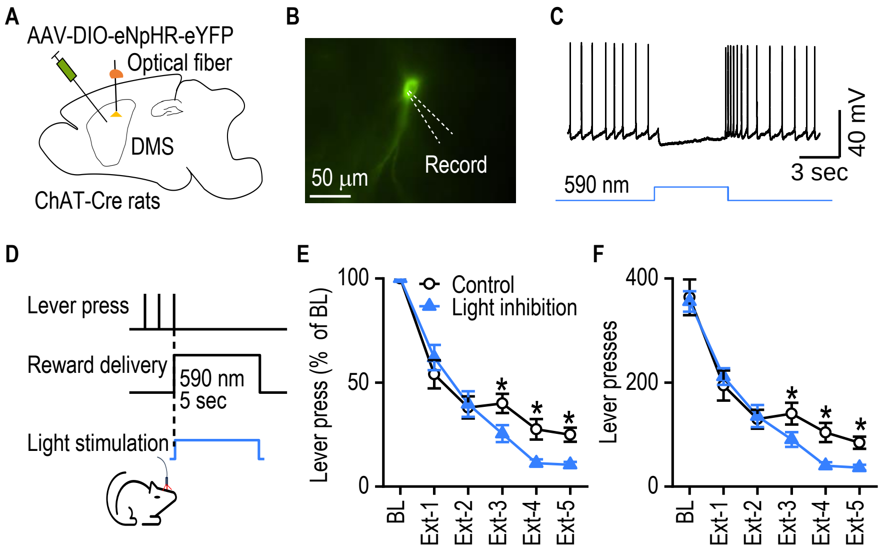

Fig. S9. Optical inhibition of CINs during extinction learning.
(**A**) Schematic of viral injection and optical fiber implantation. ChAT-Cre rats received a bilateral infusion of AAV-DIO-eNpHR-eYFP, and optical fibers were bilaterally implanted into the DMS. Rats then underwent instrumental training to receive rewards by pressing levers. (**B**) Recording of eYFP expressed CIN using whole-cell patch clamp. (**C**) Optical stimulation (590 nm, 5 sec) inhibited the firing of CIN. (**D**) Optical stimulation protocol employed during extinction training. Lever presses triggered both reward and synchronized light stimulation (590 nm) time-locked to reward delivery. The light was continuously given for 5 seconds during the reward delivery period. Actual rewards were omitted during extinction. (**E**) Lever press normalized to baseline during the extinction training. Group effect: $F_{(1,22)} = 2.68$, $p = 0.12$; session effect: $F_{(1, 4)} = 32.47$, $p < 0.001$; group x session effect: $F_{(1, 88)} = 3.75$, $p < 0.01$. (**F**) Lever presses during the extinction training. Group effect: $F_{(1, 22)} = 2.43$, $p = 0.13$; session effect: $F_{(1, 4)} = 34.91$, $p < 0.001$; group x session effect: $F_{(1, 88)} = 3.62$, $p < 0.01$. Two-way RM ANOVA followed by Tukey *post-hoc* test, $*p < 0.05$ versus control. n = 12 rats (Control) and 12 rats (Light inhibition). A 5-second light stimulation was intended to inhibit CIN firing, but it unexpectedly induced pause-rebound firing in CINs. While no significant main effect of light stimulation was observed, there was a significant interaction between light stimulation treatment and sessions. This interaction effect shows that in later sessions (Ext-3, Ext-4, Ext-5), CIN inhibition actually promoted extinction. The acceleration of extinction in later sessions could be attributed to this rebound burst firing of CINs.

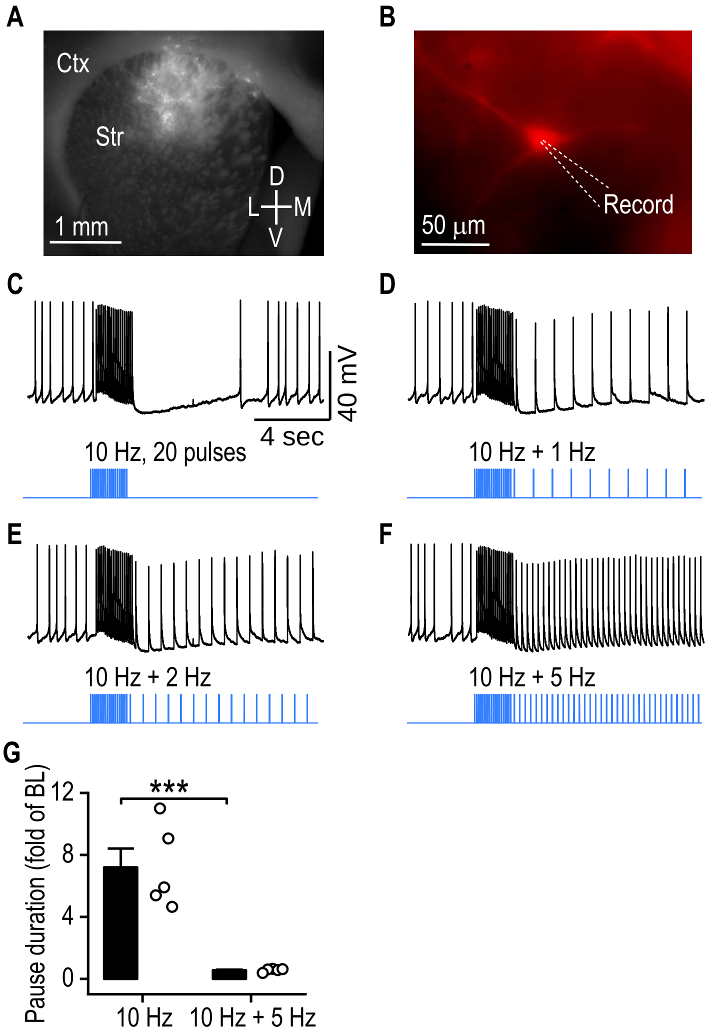

Fig. S10. Disrupting pause with continuous optical stimulations in current-clamp Recording. (**A**) The expression of AAV-FLEX-ChrimsonR-tdTomato in the DMS of ChAT-Cre rat. (**B**) Recording of tdTomato-expressing CIN using whole-cell patch clamp. (**C**) Optical stimulation (590 nm, 10 Hz, 20 pulses) induced a burst-pause firing in the CIN. Disruption of the pause in CIN firing through continuous optical stimulation with different frequencies: (**D**) 1 Hz, (**E**) 2 Hz, (**F**) 5 Hz. (**G**) Quantification of pause duration relative to baseline (BL) interevent interval showing that additional 5 Hz opto-stimulation (10 Hz + 5 Hz) abolished pause response induced by 10 Hz light stimulation; unpaired *t* test, $t_{(8)} = 5.48$, ***$p < 0.001$, n = 5 neurons for each group.

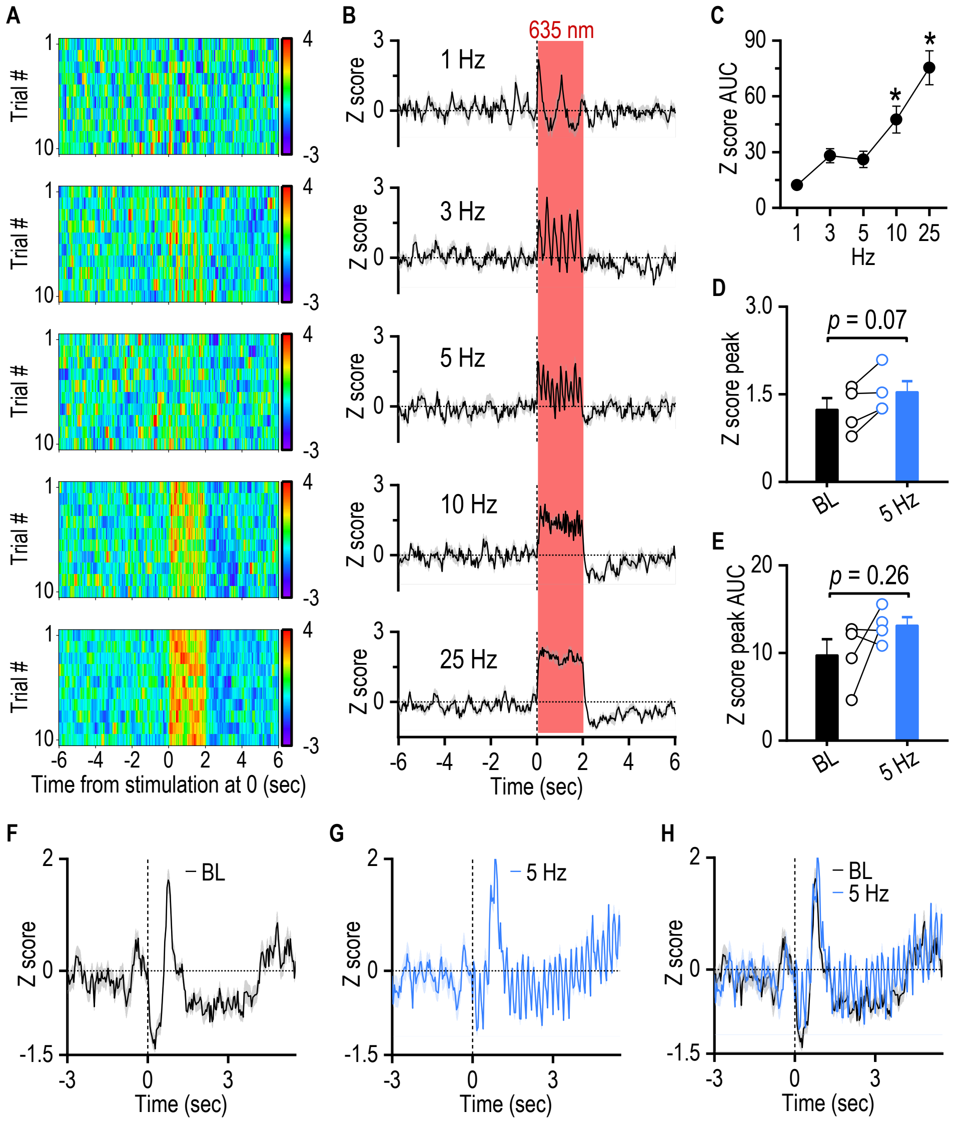

Fig. S11. ***In vivo*** **measure of ACh signal while optical stimulating CINs.**
(**A**) *In vivo* measurements of ACh signal while delivering optical stimulation to CINs (635 nm, 1 Hz, 3 Hz, 5 Hz, 10 Hz, and 25 Hz for two seconds). (**B**) Representative ACh signal traces for panel A. (**C**) Summary data quantifying the area under the curve (AUC) of ACh sensor fluorescence signals; One-way ANOVA followed by Tukey *post-hoc* test, $F_{(1, 4)} = 2.076$, $p < 0.001$, $*p < 0.05$ compared to 1 Hz stimulation. n = 4 fibers from 2 rats. (**D**) Summary data quantifying the peak ACh signal in Figure 5F; $t_{(3)} = -2.75$, $p = 0.07$ by paired *t* test, n = 4 fibers from 2 rats. (**E**) Summary data quantifying the peak AUC in Figure 5F; $t_{(3)} = -1.39$, $p = 0.26$ by paired *t* test, n = 4 fibers from 2 rats. (**F**) Raw representative ACh signal trace during operant conditioning for Figure 5F. (**G**) Raw representative ACh signal trace with 5 Hz light stimulation during operant conditioning for Figure 5F. (**H**) Overlay traces from panel F and panel G.

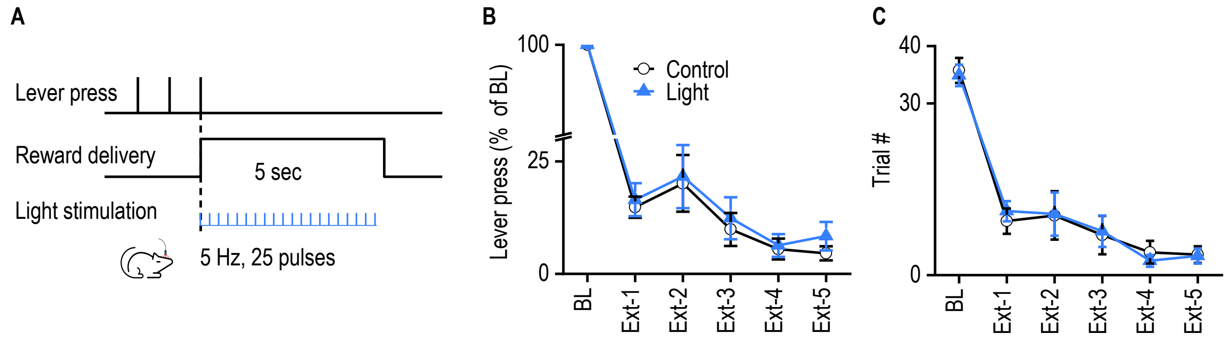

Fig. S12. Disruption of pause did not affect the extinction process.
(**A**) Optical stimulation protocol used during the extinction training. Lever presses triggered both reward delivery and synchronized light stimulation (590 nm, 5 Hz, 25 pulses for 5 sec). Actual rewards were omitted during extinction. (**B**) Lever presses during the extinction training. The data were normalized to the baseline lever presses. The two-way RM ANOVA analysis reveals no group effect ($F_{(1,11)} = 0.25$, $p = 0.63$). (**C**) The number of extinction trials. Two-way RM ANOVA shows no group effect ($F_{(1,11)} = 0.004$, $p = 0.95$). n = 7 rats (Control) and 6 rats (Light).

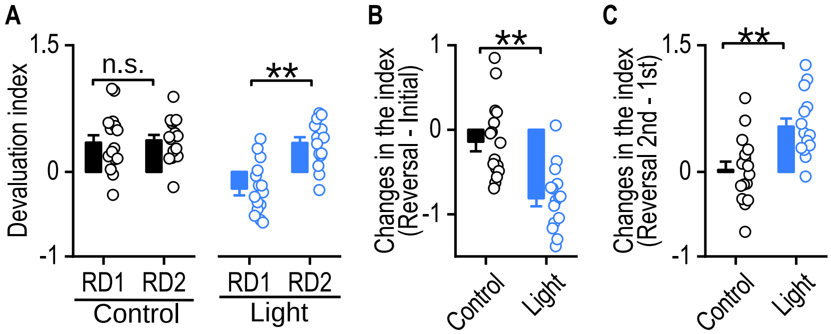

Fig. S13. Further analysis of devaluation index data of Figure 5.

(**A**) The devaluation index was not significantly different between reversal devaluation 1 (RD1) and reversal devaluation 2 (RD2) in the control group; Unpaired *t* test, $t_{(30)} = -0.24$, $p = 0.81$, n.s., not significant; the devaluation index was significantly higher in the RD2 than the RD1 for the light group, indicating a recovered reversal learning; Unpaired *t* test, $t_{(28)} = -5.13$, $^{**}p < 0.01$. (**B**) The changes in devaluation index between RD1 and initial devaluation were significantly lower in the light group than the control group, indicating an impaired reversal learning; Unpaired *t* test, $t_{(29)} = 4.47$, $^{**}p < 0.01$. (**C**) The changes in devaluation index between RD2 and the RD1 were significantly higher in the light group than the control group, indicating a recovered reversal learning; unpaired *t* test, $t_{(29)} = -3.73$, $^{**}p < 0.01$. n = 16 rats (Control) and 15 rats (Light).
